# RNA-Seq and Gene Regulatory Network Analyses Uncover Candidate Genes in the Early Defense to Two Hemibiotrophic *Colletorichum* spp. in Strawberry

**DOI:** 10.3389/fgene.2021.805771

**Published:** 2022-03-10

**Authors:** Tika B. Adhikari, Rishi Aryal, Lauren E. Redpath, Lisa Van den Broeck, Hamid Ashrafi, Ashley N. Philbrick, Raymond L. Jacobs, Rosangela Sozzani, Frank J. Louws

**Affiliations:** ^1^ Department of Entomology and Plant Pathology, North Carolina State University, Raleigh, NC, United States; ^2^ Department of Horticultural Science, North Carolina State University, Raleigh, NC, United States; ^3^ Department of Plant and Microbial Biology, North Carolina State University, Raleigh, NC, United States

**Keywords:** C*olletotrichum acutatum*, *Colletotrichum gloeosporioides*, *Fragaria × ananassa*, rate-reducing resistance, gene regulatory network

## Abstract

Two hemibiotrophic pathogens, *Colletotrichum acutatum* (Ca) and *C*. *gloeosporioides* (Cg), cause anthracnose fruit rot and anthracnose crown rot in strawberry (*Fragaria* × *ananassa* Duchesne), respectively. Both Ca and Cg can initially infect through a brief biotrophic phase, which is associated with the production of intracellular primary hyphae that can infect host cells without causing cell death and establishing hemibiotrophic infection (HBI) or quiescent (latent infections) in leaf tissues. The Ca and Cg HBI in nurseries and subsequent distribution of asymptomatic infected transplants to fruit production fields is the major source of anthracnose epidemics in North Carolina. In the absence of complete resistance, strawberry varieties with good fruit quality showing rate-reducing resistance have frequently been used as a source of resistance to Ca and Cg. However, the molecular mechanisms underlying the rate-reducing resistance or susceptibility to Ca and Cg are still unknown. We performed comparative transcriptome analyses to examine how rate-reducing resistant genotype NCS 10-147 and susceptible genotype ‘Chandler’ respond to Ca and Cg and identify molecular events between 0 and 48 h after the pathogen-inoculated and mock-inoculated leaf tissues. Although plant response to both Ca and Cg at the same timepoint was not similar, more genes in the resistant interaction were upregulated at 24 hpi with Ca compared with those at 48 hpi. In contrast, a few genes were upregulated in the resistant interaction at 48 hpi with Cg. Resistance response to both Ca and Cg was associated with upregulation of MLP-like protein 44, LRR receptor-like serine/threonine-protein kinase, and auxin signaling pathway, whereas susceptibility was linked to modulation of the phenylpropanoid pathway. Gene regulatory network inference analysis revealed candidate transcription factors (TFs) such as GATA5 and MYB-10, and their downstream targets were upregulated in resistant interactions. Our results provide valuable insights into transcriptional changes during resistant and susceptible interactions, which can further facilitate assessing candidate genes necessary for resistance to two hemibiotrophic *Colletotrichum* spp. in strawberry.

## INTRODUCTION

Plants have evolved a multilayered immune system to resist plant pathogens’ potential invasion (Jones and Dangl, 2006). Pathogen recognition through receptor complexes triggers host phosphorylation cascades and activates two types of immune signaling pathways: pattern-triggered immunity (PTI) and effector-triggered immunity (ETI) (Cui et al., 2015; Yu et al., 2017; Peng et al., 2018). PTI, triggered through the detection of non-self-microbial signatures, is the first line of inducible defense in plants called pathogen-associated molecular patterns (PAMPs) (Stotz et al., 2014). Conversely, ETI is a second layer of inducible defense, typically activated by the intracellular recognition of pathogen effector molecules by plant resistance (*R*) gene products and is often characterized by a localized hypersensitive response (HR), a type of programmed cell death (PCD) (Jones and Dangl, 2006; Cui et al., 2015). Both PTI and ETI often trigger broad-spectrum immunity to subsequent pathogen attacks, a phenomenon called systemic acquired resistance (SAR) (Fu and Dong, 2013). Another host immunity called damage-associated molecular patterns (DAMPs) was originally discovered in the mammalian immune system (Lotze et al., 2007; Boller and Felix, 2009), and later this strategy was found in plant immunity (Seong and Matzinger 2004; Lotze et al., 2007; Boller and Felix, 2009; Tanaka et al., 2014; Hou et al., 2019). PTI can be triggered by a plant’s DAMPs, which activate immune responses by perceiving host-derived molecules such as cytosolic proteins, peptides, nucleotides, and amino acids, which are released from cells undergoing pathogen invasion (Hou et al., 2019). Previous studies have shown that signaling molecules responsible for plant immunity such as receptors, kinases, and regulators of hormones play essential roles in biotrophic and hemibiotrophic interactions at early infection stages (Lenzi et al., 2016; Zuluaga et al., 2016; Becker et al., 2017, 2019). Nucleotide-binding site (NBS)–encoding genes are key plant disease resistance genes (R genes) that are responsible for plant defense against pathogen attacks (Dangl et al., 2013; Neupane et al., 2018). The NBS-encoding gene consists of three domains: a variable domain at the N-terminal, a highly conserved NBS domain in the middle, and a diverse leucine-rich repeat (LRR) domain at the C-terminal. Based on the structure of N-terminal domains, a coiled-coil (CC), Drosophila toll, and mammalian interleukin-1 receptor-like (TIR) possess resistance to powdery mildew8 (RPW8) domain. NBS-encoding genes are grouped into three subclasses, CC-NBS-LRR (CNL), TIR-NBS-LRR (TNL), and RPW8-NBS-LRR (RNL), and these proteins are responsible for the recognition of specific pathogens (Meyers et al., 2003; Qian et al., 2017; Shao et al., 2019; Xue et al., 2020; Zhang et al., 2020).

The inducible defenses and signals are associated with a wide range of transcriptional reprogramming in the plant and receptor-like kinase (RLK)–mediated immunity that may be regulated by transcription factors (TFs) (Hammond-Kosack and Jones, 1996; Li et al., 2016). Seminal work on transcriptomic profiles utilizing RNA sequencing (RNA-seq) technology (Wang et al., 2009) and streamlined bioinformatics tools have been used to identify plant defense genes and pathways specific to plant resistance at early stages of compatible and incompatible interactions in important pathosystems (Bagnaresi et al., 2012; Ah-Fong et al., 2017; French et al., 2018; Liang et al., 2018; Becker et al., 2019; McCabe et al., 2019). Comparative transcriptomic analyses revealed dynamic changes and upregulation of genes involved in defense and pathogenesis-related, signal transduction pathways, immune receptors, RLKs, TFs, hormone signaling, and lignin biosynthesis genes within members of multigene families in early cellular reprogramming during host immunity against *C. gloeosporioides* (Wang et al., 2017), *Phytophthora infestans* (Ah-Fong et al., 2017), *Magnaporthe oryzae* (Bagnaresi et al., 2012), *Leptosphaeria maculans* (Becker et al., 2019), and *Ralstonia solanacearum* (French et al., 2018).

Anthracnose fruit rot (AFR) and anthracnose crown rot (ACR), caused by *Colletotrichum acutatum* (Ca) and *C*. *gloeosporioides* (Cg), respectively, are the two major diseases of the octoploid strawberry (*Fragaria* × *ananassa,* 2*n* = 8*x* = 56) in the southeastern United States (Howard et al., 1992; Legard 2000; Peres et al., 2005; Poling 2008; Rahman et al., 2013). Recently, *C. gloeosporioides* and *C*. *acutatum* isolates have been classified as *C. gloeosporioides* species complex and *C. acutatum* species complex, respectively, based on multi-locus sequence typing (Damm et al., 2012; Weir et al., 2012; Baroncelli et al., 2015). To be consistent with our previous works, we prefer to use *C*. *acutatum* and *C. gloeosporioides* (Jacobs et al., 2019; Jacobs et al., 2020). Traditionally, the common practice to manage these diseases relied heavily upon the application of fungicides to reduce yield losses. Although planting resistant varieties is the most cost-effective and environmentally friendly approach to mitigate AFR and ACR epidemics (Peres et al., 2005; Poling, 2008), the withdrawal of several active fungicides and fumigants, and an absence of commercial varieties that exhibit a high level of resistance to multiple pathogens, are increasing challenges in strawberry production.

Like other species of *Colletotrichum*, the initial stage of infection of Ca and Cg are biotrophic (infection vesicle and primary hyphae are formed or feeding on living tissue) to necrotrophic (secondary hyphae spread within and kill the host tissue or feeding on dead tissue) phase (Perfect and Green, 2001). This sequential lifestyle development is regarded as a hemibiotrophic infection (HBI) strategy (Peres et al., 2005; Gan et al., 2013; De Silva et al., 2017). Thus, these pathogens can cause prolonged plant vegetative latent infections, multiplying without symptoms during vegetative growth stages. The high level of inoculum buildup during the vegetative stage of production raises an important question. Can strawberry-resistant varieties reduce pathogen proliferation (symptomless colonization) of vegetative tissues? If so, this will assist in developing an effective disease management strategy of the HBI to limit early cycles of sporulation.

Host plant resistance is an economical and environment-friendly method to mitigating AFR and ACR epidemics in the fruiting fields (Peres et al., 2005; Poling, 2008). Resistant cultivars with a single genetic locus or major gene are regarded as qualitative resistance to Ca and Cg (Narusaka et al., 2004; Anciro et al., 2018; Salinas et al., 2019). In contrast, quantitative (non-race specific) disease resistance (QDR) conferred by quantitative trait loci (QTL) or multiple genes is effective to multiple pathogen genera or multiple races of a pathogen (Wisser et al., 2005; Boyd et al., 2013; Corwina and Kliebenstein, 2017). Five components of disease resistance that reduce the rate of epidemics progress are infection frequency, latent period (time between the arrival of a propagule on a susceptible plant surface and the formation of the next generation of propagules), lesion size, spore production, and infectious period (period of spore production on a lesion) (Nelson, 1978; Parlevliet, 1979). Rate-reducing resistance (i.e., reduces the multiplication or spore production of the pathogen) is also known as QDR because it is often controlled by several minor genes (St. Clair, 2010) and is more durable (Johnson, 1981; Johnson, 2000) than qualitative resistance. Various lines of evidence support the multiple loci that exemplify QDR confer resistance to bacterial wilt caused by *Ralstonia solanacearum* in tomato (Carmeille et al., 2006; Wang et al., 2013), leaf blight incited by *Cochliobolus heterostrophus* in maize (Poland et al., 2009), and bacterial blight caused by *Xanthomonas oryzae* pv. *oryzae* in rice (Bossa-Castro et al., 2018). Importantly, resistance to *R. solanacearum* is mediated by RRS1-R, a gene encoding TIR-NBS-LRR protein (Deslandes et al., 2002; Deslandes et al., 2003). However, little is known about interactions between rate-reducing resistance in strawberry and two hemibiotrophic *Colletotrichum* spp.

North Carolina State University is one of the leading institutions for strawberry breeding in the USA. It provides unique certified pathogen-free genetic resources through micropropagation and the National Clean Plant Network (NCPN). One of the North Carolina advance selections, NCS 10-147 exhibited high levels of rate-reducing resistance to both Ca and Cg in our previous studies (Jacobs et al., 2019; Jacobs et al., 2020). Our findings suggested that the colonization (i.e., spore production) of leaf tissue by Ca and Cg, disease severity, and area under disease progress curves (AUDPC) were significantly reduced in the resistant genotype NCS 10-147 (Jacobs et al., 2019). We hypothesize that the genetic expression of the rate-reducing resistance to two hemibiotrophic *Colletotrichum* spp. may be implicated by triggering the early transcriptional reprogramming events and eventually modulating plant defense to mitigate AFR and ACR epidemics in the fruiting field (Jacobs et al., 2019; Jacobs et al., 2020). To date, biphasic interactions (biotrophic and hemibiotrophic stages) of Ca and Cg with rate-reducing resistance in strawberries remain an intriguing question.

We hypothesized that both Ca and Cg go through HBI and have a close interaction with the host during the early active phase of pathogen establishment and may modulate plant defense responses at the infection site of leaf tissues. In this study, we leveraged the RNA-seq (Wang et al., 2009) and gene regulatory network (GRN) (Van den Broeck et al., 2020) analyses to uncover the distinct genes associated with the early plant defense or susceptibility to two hemibiotrophic *Colletotrichum* spp. and identify key regulators associated with target genes (Ko and Brandizzi, 2020). Understanding of strawberry–Ca and Cg HBI interactions in response to active phases of these pathogens can ultimately advance in developing resistant strawberry cultivars in the Southeastern United States where AFR and ACR epidemics cause significant yield losses.

## MATERIALS AND METHODS

### Selection of Strawberry Genotypes

*Fragaria × ananassa*, the advanced breeding selection, NCS 10-147 developed from a cross between ‘Treasure’ and ‘Chandler’ exhibited a high level of resistance to both Ca and Cg HBI (Jacobs et al., 2019; Jacobs et al., 2020) and was selected as the resistant genotype for this study. Commercial variety ‘Chandler’ (Douglas × Cal 72.361-105) is a high-yielding commercial variety and extensively grown in the Southeastern United States (Chandler, 1991; Poling, 1993), but it is susceptible to both Ca and Cg (Poling, 1993; Jacobs et al., 2019).

### Plant Production and Plant Growth Conditions

Six disease-free mother plants of resistant genotype NCS 10-147 and susceptible cv. ‘Chandler’ were planted in pots and grown to maturity on drip irrigation in a greenhouse in Kannapolis, NC. Clean runners were collected from mother plants and processed to yield 45 clonal tips for each genotype. Tips were planted in 50-cell plug trays containing Fafard 3B potting mix and were rooted under intermittent mist (30-s duration at 10-min interval) in a 21 ± 5°C greenhouse with a 12 h light/dark photoperiod for 1 week until roots had developed. Rooted plug plants were potted into 4″ pots containing Fafard 3B potting mix and were overwintered in a 19 ± 5°C greenhouse. The plants were hand-watered daily and fertilized with a solution of 15:5:15 (N-P-K) fertilizer weekly (Jack’s Professional LX Ca-Mg, 100 ppm N; JR Peters, Inc., Allentown, PA) *via* a Dosatron injector system (Dosatron International, Inc., Clearwater, FL). Plants received overhead supplemental lighting (fluorescent, 11,000 lux) for 3 weeks before inoculation to increase the day length to 14 h and encourage vegetative growth.

### Inoculum Preparation and Inoculation

The isolates of Ca and Cg originally collected from Castle Hayne, NC (Jacobs et al., 2020) were used for the inoculation. These isolates were revived on acidic potato dextrose agar (A-PDA; BD Diagnostic Systems, Sparks, MD) plates. The plates were incubated for 10 days at 25°C under 12 h fluorescent light. Conidia were harvested by flooding the mycelium with distilled water containing Tween 20 (20 µl/100 ml) (polyoxyethylenesorbitan monolaurate; Sigma-Aldrich, St. Louis, MO) and disturbing the mycelium with a glass stirring rod to suspend conidia. Conidial suspension of Ca and Cg passed through a doubled layer of cheesecloth to remove cellular debris and the inoculum concentration of each isolate was adjusted to 1.0 × 10^6^ conidia/ml using a hemacytometer. Leaves on separate plants were spray inoculated with Ca or Cg using a handheld sprayer (Solo model 419) until runoff. Plants inoculated with distilled water served as (mock) controls. After inoculation, the test plants were left in greenhouse chambers at a 48-h incubation period of intermittent mist (3-s duration at 5-min interval) and temperatures raised to 25°C to promote disease establishment. After 48-h misting, the plants were left at 25 ± 1°C throughout the experiment.

### Time-Course Experiments

The experiments were laid out in a split-plot design where pathogen and mock (water) as controls were the main plots and strawberry genotypes were subplots and conducted two times on greenhouse benches. Each experiment had three replications using each bench as a block. To identify differentially expressed genes (DEGs) in leaf tissues of the resistant genotype NCS 10-147 and the susceptible genotype ‘Chandler’, the interactions between each genotype and Ca and Cg were investigated at 0, 24, and 48 post-inoculation (hpi) with Ca and Cg independently and mock inoculation (Supplementary Figure S1). Tri-foliate leaves per plant were used as the experimental unit. Among these, one leaf for each treatment regimen (genotype—pathogen—timepoint) was selected for DNA extraction, RNA isolation, and paraquat assays. The inoculated leaves from each plant were collected separately and frozen in liquid nitrogen immediately after harvesting.

### Disease Assessment

We used paraquat assays to investigate leaf tissue colonization by Ca and Cg in the resistant genotype NCS 10-147 and susceptible genotype ‘Chandler’. Herbicide paraquat (1,1′-dimethyl-4′4-bipyridinium dichloride) (Mertely and Legard, 2004) was used to induce spore production of both Ca and Cg on symptomless leaf tissues of both genotypes at 0, 24, and 48 hpi. Mock inoculation was used as a control. Briefly, six leaflets of each genotype for each timepoint were surface sterilized with immersion in 70% EtOH for 15 s, 10% bleach for 60 s, and rinsed twice in sterile distilled water. Leaflets were then immersed for 1 min in a solution of 0.20% paraquat and rinsed one final time in sterile distilled water before being placed in plastic bags lined with a double layer of sterile paper towels. The prepared sample bags were incubated for 4 days at 25°C. After 7 days of incubation, colonization of leaves showing any visible growth of salmon color acervuli (a small asexual fruiting body that erupted through the epidermis of leaf surfaces) was assessed using computer-guided imaging software ImageJ (Abd-El-Haliem, 2012). Disease severity data (percentage of sporulating leaf area) from the greenhouse experiments were collected using Horsfall and Barratt’s disease grading system (Horsfall and Barratt, 1945). These data were analyzed to compare the resistance in NCS 10-147 and ‘Chandler’ having incompatible and compatible interactions to Ca and Cg using Statistical Analysis System (SAS) ver.9.4 (SAS Institute Inc., 2019). Statistically significant differences were assumed at *p* ≤0.01. To confirm their identity, spores of Ca and Cg developed on paraquat-treated leaves of both genotypes were isolated on A-PDA medium and identified by quantitative real-time PCR (qPCR) as previously described (Garrido et al., 2009).

### Total RNA Extraction

Six leaflets were collected from each genotype at 0, 24, and 48 hpi with Ca and Cg, and mock inoculation and pooled for each timepoint in each replicate. Total RNA was isolated from freeze-dried leaf tissues of each sample using the Qiagen RNeasy Plant Mini Kit (Qiagen, Germantown, MD). The RNA samples were further treated with DNase (Promega, Madison, WI), eluted in RNase-free water, and quantified using a NanoDrop 2000 spectrophotometer (Thermo Fisher Scientific). The RNA integrity was analyzed using an RNA Nano 6000 Assay Kit of the Agilent 2100 Bioanalyzer system (Agilent Technologies, Santa Clara, CA). The extracted RNAs (50 µg) from leaf samples of two independent experiments were pooled for each genotype at each timepoint. The RNA from each sample was resuspended in 50 µl of nuclease-free water (Thermo Fisher Scientific, Waltham, MA).

### Library Preparation and Sequencing

The cDNA libraries were constructed using the NEBNext Ultra Directional RNA Library Prep Kit (New England Biolabs, Ipswich, MA). The cDNA libraries were constructed using a Truseq RNA Library Preparation Kit (Illumina, San Diego, CA). In brief, mRNA isolation, fragmentation, and priming were performed using the NEBNext Poly(A) mRNA Magnetic Isolation Module (NEB #E7490). After cDNA preparation, adaptors and individual barcodes were added through PCR amplification and paired-end 100-bp reads were performed on all samples using an Illumina Hi Seq 2500 platform at the Genomic Sciences Laboratory (GSL), North Carolina State University, Raleigh, NC. All libraries were prepared simultaneously, and all samples were sequenced in the same run.

### RNA-seq Data Processing

Quality processing was performed using trim_galore ver. 6.0, a wrapper around cutadapt and afterQC ver.9.3 (Chen et al., 2017). First, adapter trimming and quality filtering were performed using default settings on trim_galore. The trim_galore output was further curated using afterQC to reads containing homopolymers and low-quality nucleotides. The afterQC filtering parameters were set as follows: `--poly_size_limit 25 -- allow_mismatch_in_poly 5 -- seq_len_req 25 -- trim_front -1 -- trim_tail -1`. Finally, reads shorter than 25 bp after quality and adapter trimming were discarded.

### Transcriptome Assembly

*De novo* assembly of the RNA-seq reads was performed on the CLC-genomics workbench ver. 10 from Qiagen^1^. Twenty-four independent *de novo* assemblies were generated from the clean RNA-seq reads using different assembly parameters (word size: 30, 31, 32, 33, 34, and 35; bubble size: 250, 300, 350, and 400; and minimum contig length of 300 nt). The best assembly with the largest N50 (N50 = 849 nt) and the lowest number of contigs (*n* = 77,883) was obtained from the word size (k-mer) 31 and bubble size 300 parameters and was used as the final assembly for further downstream analysis of differential gene expression.

### Elimination of Ca and Cg Transcripts and Mapping of mRNA-seq Data

All short single-end reads were mapped to the reference genome of Ca strain 1^2^ and Cg strain 14^3^ with TopHat ver. 2.0.6 (Trapnell et al., 2012). After excluding Ca and Cg genes from reference annotations, we mapped all clean reads to the strawberry reference genomes (Shulaev et al., 2011; Edger et al., 2019) using TopHat ver. 2.0.6 (Trapnell et al., 2012). These contigs were further blasted using USEARCH (Edgar, 2010) to a protein database consisting of protein sequences of *Fragaria vesca* (Shulaev et al., 2011) and *Fragaria × ananassa* (Edger et al., 2019). Based on the BLAST output, if multiple transcripts had the exact hits, they were merged using BLAST2CAP3^4^. Hierarchical clustering analysis was used to determine the effect of treatments (pathogen *vs* mock inoculation, or resistant *vs* susceptible interaction) for all samples using variance stabilization transformation for the read count data (Ge et al., 2018). In addition, clustering was based on Z scores, which measure the number of SDs relative to the mean for all samples. Euclidian distances were estimated for all samples, and an Unweighted Pair Group Method with Arithmetic Mean (UPGMA) and heat maps were generated using heatmaps (Kolde, 2013). Gene expression levels were analyzed by Pearson’s correlation analyses of log2 fragments per kilobase of transcript per million fragments mapped (FPKM) (Ge et al., 2018).

### Differential Gene Expression Analysis

DEGs were identified using the Trinity RNA-seq analysis pipeline (Haas et al., 2013). The Trinity pipeline was set to use the RNA-seq by Expectation-Maximization (RSEM) method for transcript abundance estimation. The DESeq2 package^5^ ver. 3.14.0 was used for differential expression estimation (Love et al., 2014). Pairwise comparisons were made between the transcripts of each pair of genotypes, timepoints, and treatments. The significant difference was determined using a false discovery rate (FDR) <0.05 and the absolute value of log2 fold change (FC) ≥ |1|. For time-course analysis, we used mock-inoculated leaves at 0 hpi as a standard baseline. Resistant genotype or susceptible genotype inoculated with Ca and Cg at 0, 24, and 48 hpi was compared with mock 0 h to identify DEGs in response to Ca and Cg. The data of each lane were analyzed separately and combined by sample using an Integrated Differential Expression and Pathway (iDEP)^6^ analysis that connects 63R/Bioconductor packages, two web services (Ge et al., 2018).

### Gene Ontology and Pathway Enrichment Analysis

GO terms were derived from annotations of the sequenced strawberry reference genomes (Shulaev et al., 2011; Edger et al., 2019) using BLAST2GO software package ver. 2.0 (Götz et al., 2008). GO terms enriched in the set of the DEGs either at a single timepoint or at combined timepoints were identified using GO: TermFinder (Boyle et al., 2004). To identify significant DEGs, the absolute value of log2 FC ≥ |1| and false discovery rate (FDR) *p* < 0.05 were calculated using DESeq2 ver. 3.14 (Love et al., 2014). Significantly upregulated and downregulated GO terms were grouped into three categories, biological processes, molecular functions, and cellular components. Strawberry genes were identified as upregulated or downregulated in resistant and susceptible interactions with Ca and Cg relative to mock inoculation and compared between the resistant and the susceptible interactions at the corresponding timepoint. GO figures were generated using the Multiple Experiment Viewer from TM4 (Saeed et al., 2003; 2006). Kyoto Encyclopedia of Genes and Genomes (KEGG), which is a database resource^7^ that integrates genomic, chemical, and systemic functional information (Kanehisa et al., 2017), was used to annotate metabolic pathways and enzymes. To identify the significantly enriched metabolic pathways, signal transduction pathways, and enzymes, the FC values for all genes generated by DESeq2 Ver. 3.14.0 were analyzed using Gene Set Enrichment Analysis (GSEA) (Conesa et al., 2005; Subramanian et al., 2005). Furthermore, TFs were identified using the strawberry reference genome (Edger et al., 2019). Comparisons were made between the pathogen-inoculated at 0, 24, and 48 hpi with the mock-inoculated timepoint at 0 h, and between the pathogen-inoculated resistant and susceptible interactions at each timepoint 0, 24, and 48 hpi. To identify significantly downregulated pathways and enzymes, susceptible interactions were compared with resistant interactions at 24 and 48 hpi, and the count matrix was imported into the R graphics program using ggplot2 ver. 2.0 (Wickham, 2009).

### Validation of Selected DEGs by qPCR

To validate DEGs identified by RNA-seq analysis, 10 upregulated and downregulated genes such as defense genes (strawberry CLC_DN25_c464_g464, strawberry CLC_DN25_c1204_g1204), signal transduction (strawberry CLC_DN25_c43985_g43985, strawberry CLC_DN25_c9644_g9644, strawberry CLC_DN25_c12968_g12968), TFs (strawberry CLC_DN25_c21246_g21246, strawberry CLC_DN25_c4215_g4215, strawberry CLC_DN25_c4058_g4058), lipid metabolism (strawberry CLC_DN25_c475_g475), and hormone signaling (strawberry CLC_DN25_c5560_g5560) were quantified by qPCR (Supplementary Table S1). Primers of targeted all genes were designed from the octoploid strawberry reference genome cv. ‘Camarosa’ (Edger et al., 2019) and located on different chromosomes in the homologous region of the sub-genomes. Primer sequences were assembled using the Primer3 software package (Rozen and Skaletsky, 2000), with primer lengths ranging from 18 to 24, and amplified product lengths of 83–127 bp. The glyceraldehyde 3-phosphate dehydrogenase (*GAPDH*) (Amil-Ruiz et al., 2013) was selected and used as an internal control. An aliquot of the same RNA sample used for the RNA-seq libraries was used for cDNA construction and qPCR experiment. Gene expression (relative fold change to reference gene) was quantified using the Relative Standard Curve method (Livak and Schmittgen, 2001). SDs (*p* ≤ 0.01) were calculated for both resistant and susceptible interactions.

### Gene Regulatory Network Analysis

The strawberry (*Fragaria vesca*) TFs were downloaded^8^. In all, 1,250 coding sequences (CDS) of strawberry TFs (Supplementary Figure S2) were used to align them against the entire transcriptome (Supplementary Table S2) using the Genomic Mapping and Alignment Program (GMAP, V2015-07-23) software package with different parameters (Wu and Watanabe, 2005). Since the TF sequences were coding sequence derived, we applied the “no splice” option. No initial mapping stringencies were applied to determine the minimum percent identity (MPI = 0.0) and the minimum trimmed coverage (MTC = 0.0) to establish the query length and alignment. Subsequently, we increased the MPI and MTC to 95% for both parameters and compared them. To obtain a GRN and identify the causal relationships between genes, DEGs were identified in the pairwise comparison at 0–24 hpi and 0–48 hpi using FDR <0.05 and the log2FC ≥ |1| as our selection criteria. These pairwise comparisons were performed for both interactions, ‘Chandler’ (susceptible) and NCS 10-147 (resistant), and treatments, Ca, and Cg. This resulted in the identification of 947 and 1,728 DEGs after Ca and Cg infection in ‘Chandler’ and 522 and 740 DEGs after Ca and Cg infection in NCS 10-147 for all two timepoints combined. Next, the common DEGs between the two treatments for each genotype were identified and used as input gene lists for network inference. In total, 741 and 318 DEGs were selected for ‘Chandler’ and NCS 10-147, respectively. To generate the GRN, a regression tree–based inference algorithm was used for network inference on each of the two sets of DEGs (Clark et al., 2019; Spurney et al., 2020). To this end, a MATLAB graphical user interference (GUI), TuxNet^9^ was used. To employ regression tree inference and predict causal regulation, 10 iterations were performed. All networks were visualized in Cytoscape ver. 3.8.0 (Lopes et al., 2010).

## RESULTS

### Disease Assessment

**FIGURE 1:**
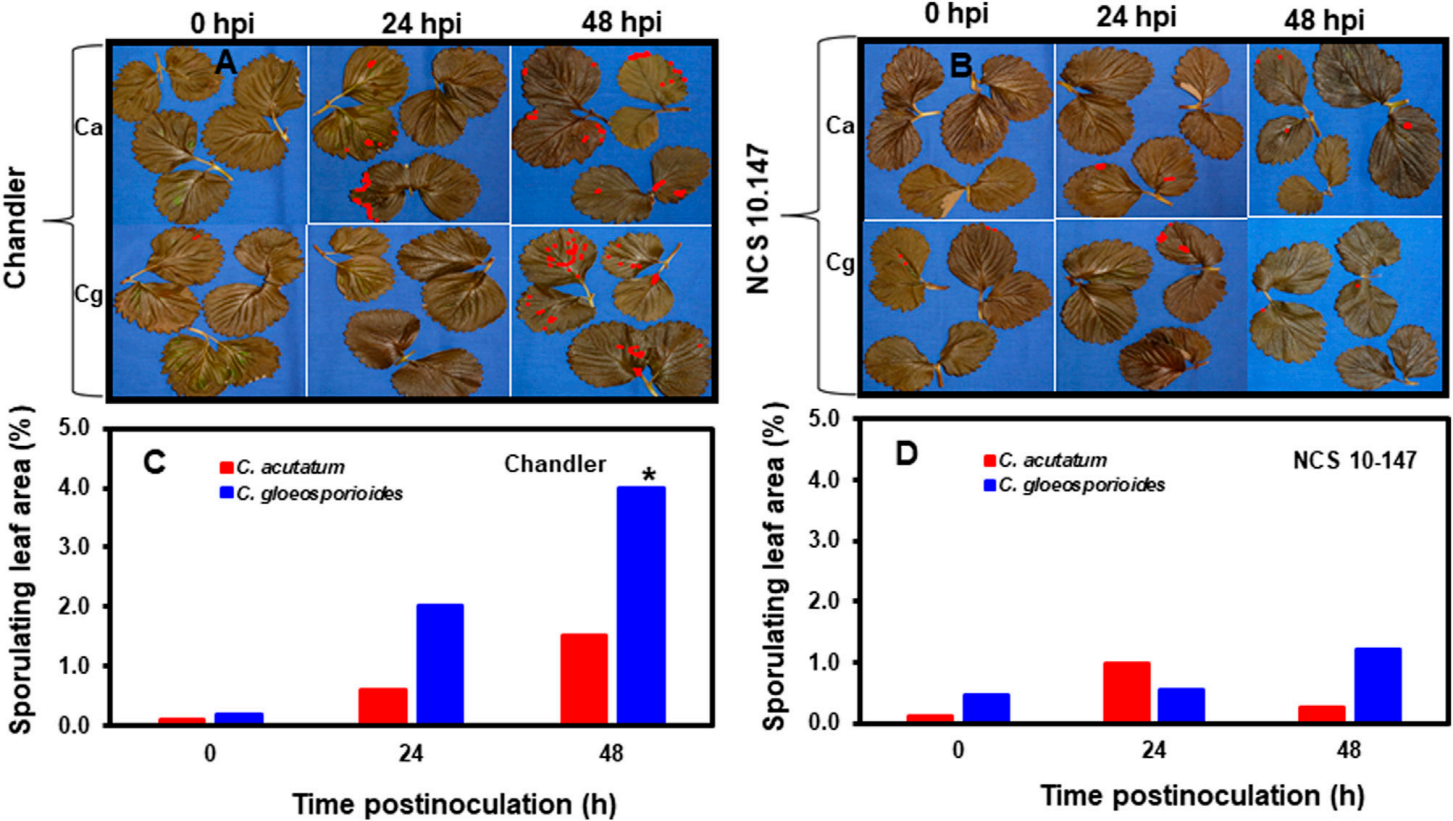
The development of anthracnose disease on strawberry leaves. **(A)** Visualization of acervuli in inoculated and paraquat-treated leaves in the susceptible genotype ‘Chandler’, and **(B)** the resistant genotype NCS 10-147 at 0, 24, and 48 h post-inoculation (hpi) using image analysis. Percentage sporulating leaf area using image analysis **(C)** in the susceptible interactions and **(D)** in the resistant interactions at 0, 24, and 48 hpi using image analysis. Asterisk (*) indicates significant differences at *p* ≤ 0.01.

Acervuli formation and necrotic lesions in the inoculated leaves was not observed at 0 hpi in resistant and susceptible interactions (Figures 1A,B). Although acervuli formation and colonization of the leaf surface were observed between 24 and 48 hpi for Ca and Cg, differences were noted between resistant and susceptible plant leaf tissues during acervuli formation of both Ca and Cg at each timepoint. In leaf tissues of the susceptible genotype ‘Chandler’, acervuli formation was higher at 48 hpi than at 24 hpi. The percentage of sporulating leaf area was calculated to compare early plant responses to Ca and Cg. Compared with Ca, Cg caused a significantly (*p* ≤ 0.01) higher percentage of sporulating leaf area in the susceptible plants at 48 hpi (Figures 1C,D). Low sporulating leaf areas developed in the resistant genotype NC-10-147 at 0, 24, and 48 hpi with Ca and Cg, presumably due to the reduction of acervuli formation (an indirect indicator of fungal colonization) and fungal penetration. No significant differences in the percentage of sporulating leaf area were observed between Ca and Cg in the resistant genotype at any timepoint.

### Discovery of Strawberry Transcriptomes in the Early Infection in Resistant and Susceptible Interactions

**FIGURE 2:**
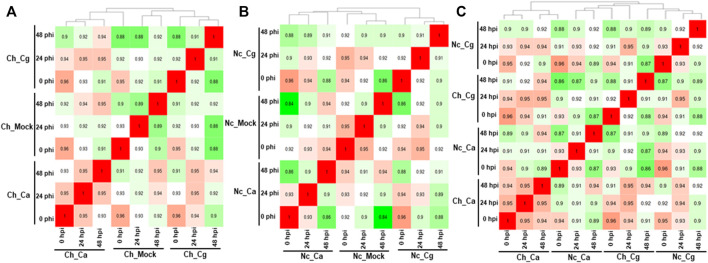
Pearson’s correlation values were calculated for all pair-wise comparisons using the clustering method in resistant interactions and susceptible interactions with *Colletotrichum acutatum* (Ca) and *C. gloeosporioides* (Cg). In total, 77,883 transcripts were identified after fulfilling the selection criteria. **(A)** Susceptible interactions when compared with mock. **(B)** Resistant interactions when compared with mock. **(C)** Resistant interactions when compared with susceptible interactions.

**FIGURE 3:**
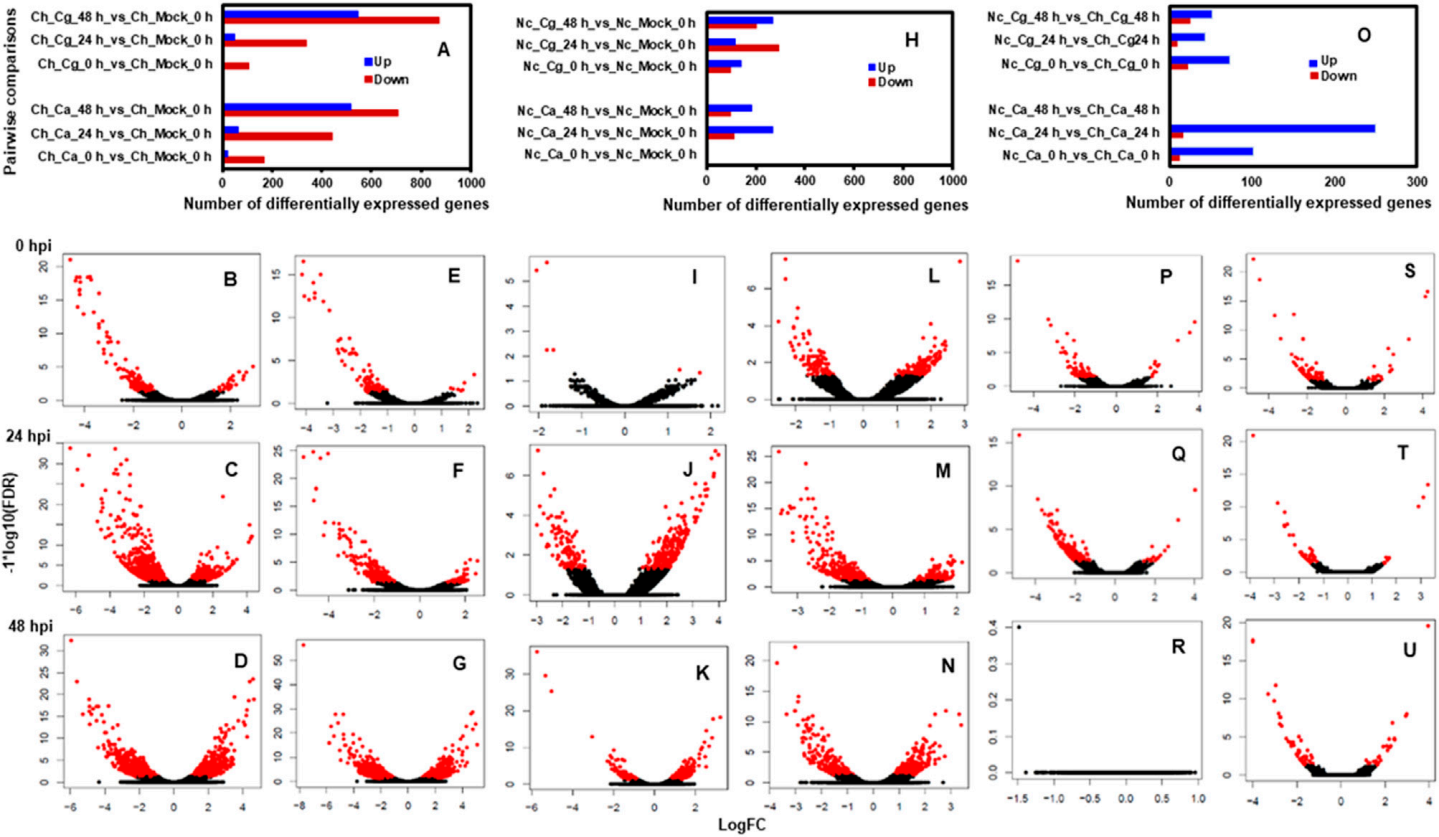
Pairwise comparisons during resistant interactions and susceptible interactions in response to *Colletotrichum acutatum* (Ca) and *C. gloeosporioides* (Cg). Water (mock) spray-inoculated leaves served as controls. Mock treated leaves at 0 hpi was considered a standard baseline. Resistant and susceptible interactions with Ca and Cg at 0, 24, and 48 hpi were compared with mock at 0 hpi to identify differentially expressed genes (DEGs). The threshold for differential expression was calculated to log2 fold change (FC) ≥ |1| with a false discovery rate at *p* < 0.05. DEGs are shown with red dots, while non-DEGs are in black. The log2FC values greater than 0 indicate upregulated (right side) and less than 0 indicate downregulated (left side) genes. **(A)** Numbers of DEGs during susceptible interactions when compared with mock at 0 hpi. **(B–G)** Volcano plots of gene expression profiles were obtained during susceptible interactions in response to Ca and Cg. **(H)** Numbers of DEGs during resistant interactions in response to Ca and Cg when compared with mock at 0 hpi. **(I–N)** Volcano plots of gene expression profiles during resistant interactions. **(O)** Numbers of DEGs during resistant interactions when compared with susceptible interactions at 0, 24, and 48 hpi. **(P–U)** Volcano plots of gene expression profiles during resistant interactions when compared with susceptible interactions.

Approximately 13 to 15 million pairs of filtered base reads were obtained for each replicate (*data not shown*). In all, 77,883 transcripts were assembled after applying filtering criteria (Supplementary Table S2). Pearson’s correlation coefficients of DEG values ranged from 0.86 to 0.96 (Figures 2A–C), indicating a good level of reproducibility among biological replications within the treatment. To gain insights into the transcriptional responses, we compared pathogen-inoculated treatments at 0, 24, and 48 hpi with mock. In susceptible interaction, 190, 504, and 1,228 DEGs were responsive to Ca, whereas 114, 385, and 1,416 DEGs were responsive to Cg at 0.24 and 48 hpi, respectively (Figure 3A). In general, more DEGs (57.74–87.89% of the total DEGs and 61.58–93.86% of the total DEGs) were downregulated in susceptible interactions at 48 hpi with both pathogens when compared with mock at 0 and 24 hpi, respectively (Figures 3B–G). In contrast, more DEGs were upregulated in resistant interactions with Ca at 0, 24, and 48 hpi (Figures 3H–K). Similarly, 58.9, 28.54, and 56.87% DEGs were upregulated, and 41.1, 71.46, and 43.13% were downregulated with Cg at 0, 24, and 48 hpi, respectively (Figures 3H,L–N). We also compared the DEGs in resistant and susceptible interactions at 0, 24, and 48 hpi with mock at 0 hpi. In general, a greater abundance of DEGs was upregulated at 24 hpi compared with that at 0 and 48 hpi (Figure 3O), but no DEGs were detected at 48 hpi (Figures 3P–U).

**FIGURE 4:**
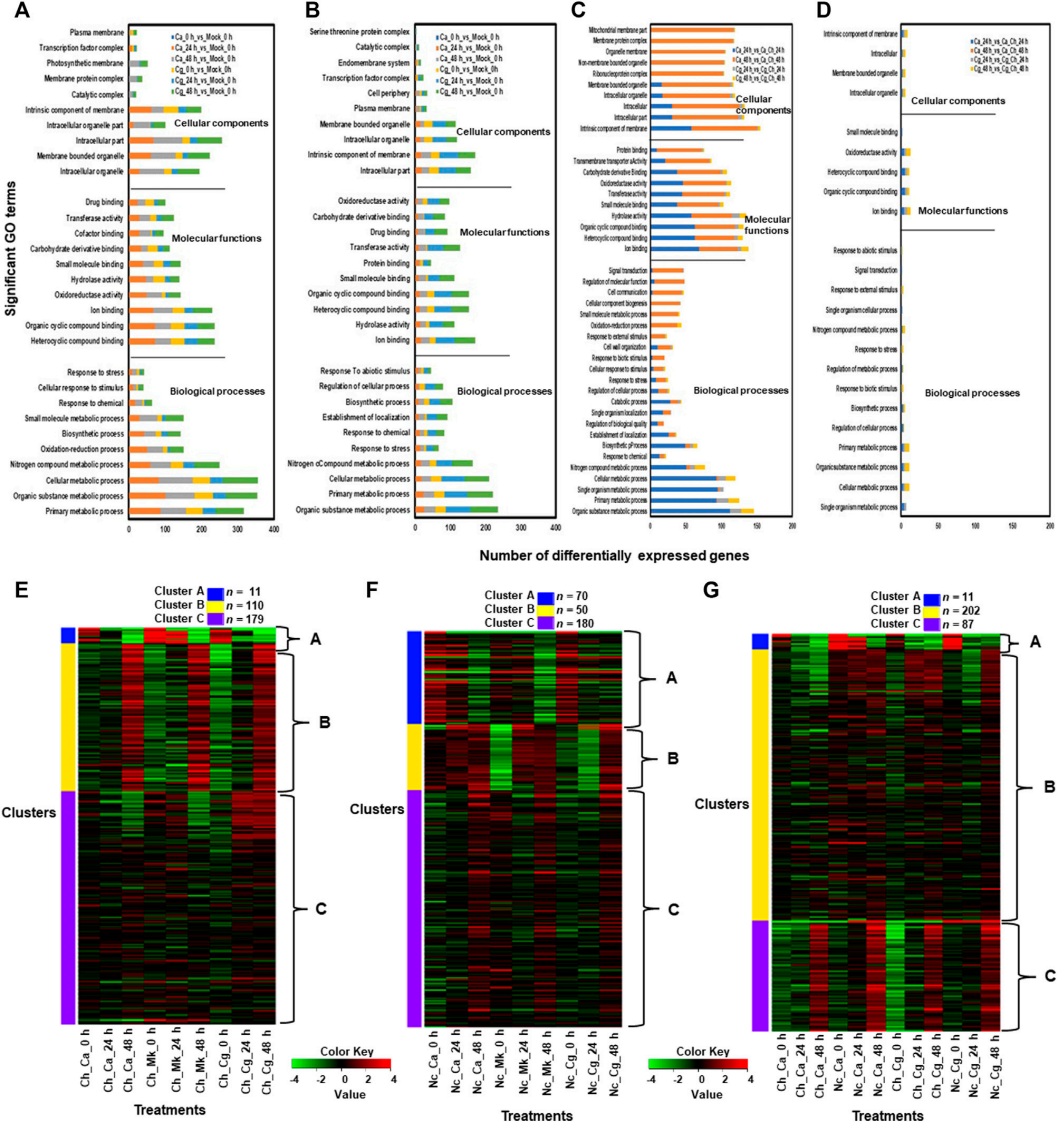
Gene ontology (GO) terms related to upregulated and downregulated genes in each treatment. We used mock (water) treated leaves at 0 h post-inoculation (hpi) as a standard baseline. Resistant and susceptible interactions with *Colletotrichum acutatum* (Ca) and *C. gloeosporioides* (Cg) at 0, 24, and 48 hpi were compared with mock at 0 hpi. The absolute value of log2 fold change (FC) ≥ |1| and false discovery rate *p* < 0.05 were calculated. **(A)** A set of GO terms related to upregulated genes during resistant interactions with Ca and Cg when compared with mock at 0, 24, and 48 h post-inoculation (hpi). **(B)** A set of GO terms related to downregulated genes during resistant interactions with Ca and Cg when compared with mock. **(C)** A set of GO terms related to upregulated genes during resistant interactions with Ca and Cg when compared with susceptible interactions with Ca and Cg. **(D)** A set of GO terms related to downregulated genes during resistant interactions with Ca and Cg at 24 and 48 hpi when compared with susceptible interactions with Ca and Cg at 24 and 48 hpi. **(E–G)** Based on hierarchical clustering analysis, the top 300 DEGs during resistant and susceptible interactions at 0, 24, and 48 hpi were selected and grouped into three clusters. Gradient colors indicate the changes in the expression level of each gene. A Z-score with green indicates a decrease and red refers to an increase in gene expression level.

Molecular functions related to the top five upregulated GO terms in resistant interactions were heterocyclic compound binding, organic cyclic compound binding, iron-binding, oxidoreductase activity, and intracellular organelle genes (Figure 4A). The cellular components consisted of the membrane-bound organelle, intracellular organelle, intracellular part, and intrinsic elements of membrane genes activated in cellular compartments. The biological processes related to five significantly upregulated GO terms were organic substance metabolic processes, primary metabolic process, cellular metabolic process, nitrogen compound metabolic process, and responses to stress genes (Figure 4B). GO terms related to molecular functions significantly downregulated by both Ca and Cg were ion binding, hydrolase activity, heterocyclic compound binding, organic cyclic compound binding, and small molecule binding at each timepoint. In contrast, cellular components related to five significantly downregulated GO terms in the resistant interactions were an intracellular part, intracellular, intrinsic elements of the membrane, intracellular organelles, and membrane-bound organelles. A higher number of GO terms were unregulated in resistant interactions compared with susceptible interactions (Figures 4C,D), suggesting that various biological processes and cellular defense mechanisms are engaged in resistant interactions in response to both Ca and Cg. Although GO terms related to response to biotic and abiotic stimuli, stress response, and signal transduction were overrepresented in susceptible interactions, Ca downregulated more genes at 24 hpi than Cg. Although marked differences in GO terms regulation were detected between resistant and susceptible interactions, we found a similar trend of upregulated and downregulated GO terms in response to both Ca and Cg at different timepoints and the plant responded similarly to both Ca and Cg (Supplementary Figure S3A–F, S4A–F). Based on hierarchical clustering analysis, the top 300 DEGs across three timepoints were selected and grouped into three clusters. When susceptible interaction was compared with mock, 11, 110, and 179 DEGs were present in clusters A, B, and C, respectively (Figure 4E). Similarly, when resistant interaction compared with mock, 70, 50, and 180 DEGs were consisted of clusters A, B, and C, respectively (Figure 4F). In comparisons between resistant and susceptible interactions, clusters A, B, and C contained 11, 202, and 87 DEGs, respectively (Figure 4G).

### Upregulation of Plant Defense Genes in Resistant Interactions

**FIGURE 5:**
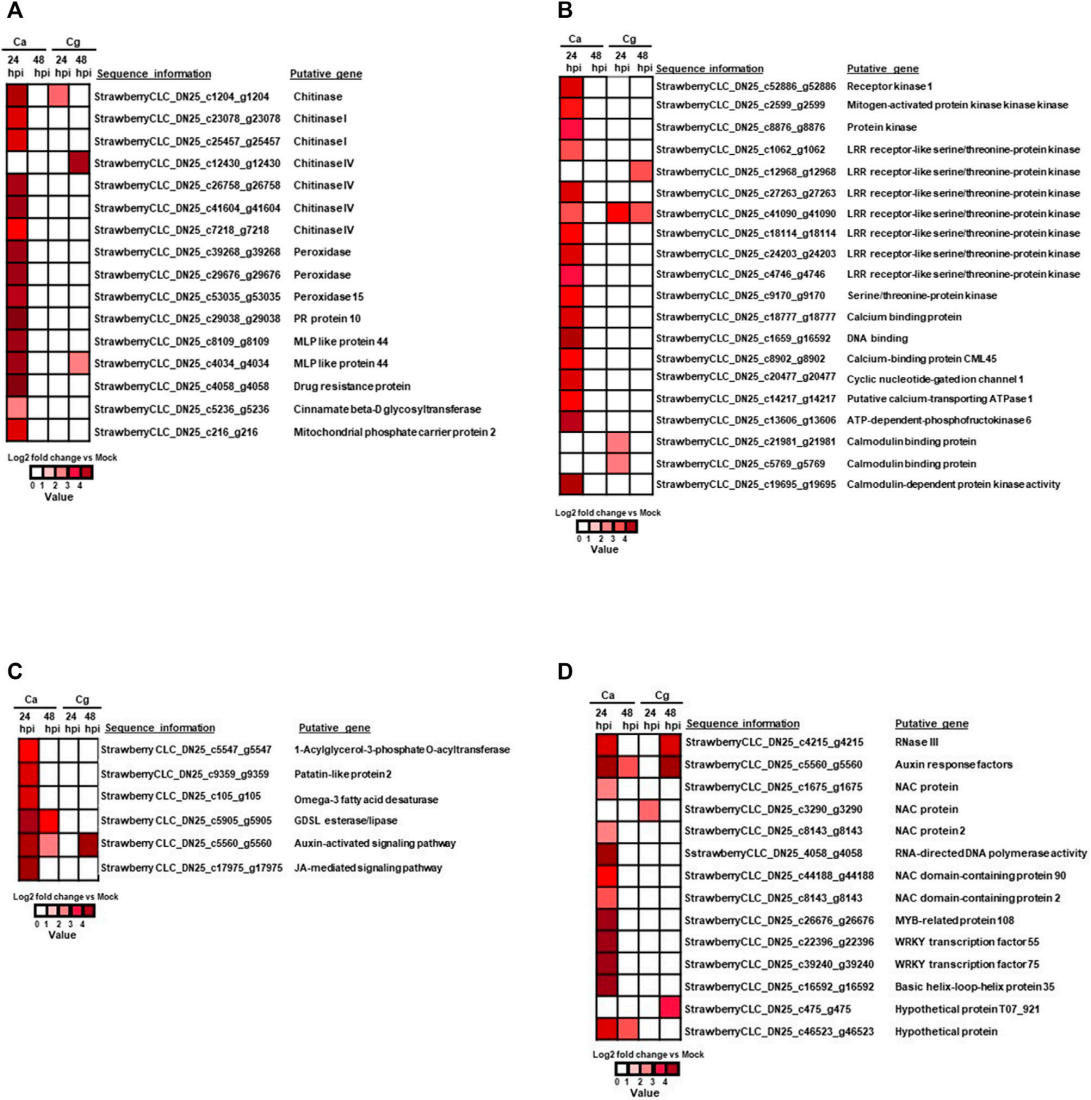
Heatmap of differentially expressed genes (DEGs) during resistant interactions at 24 and 48 h post-inoculation (hpi) in response to *Colletotrichum acutatum* (Ca) and *C. gloeosporioides* (Cg) when compared with mock at 0 h. Relative log2 fold change ≥ |1| with a false discovery rate *p* < 0.05. The brightness of red color as indicated by the gene expression value in each rectangular is from low to high. **(A)** Plant defense–related genes upregulated in response to Ca and Cg at 24 and 48 hpi. At 48 hpi, no genes were differentially upregulated in response to Ca. **(B)** Immune receptors and signal transduction-related genes. At 48 hpi, no genes were differentially upregulated in response to Ca. **(C)** Lipid metabolism and hormone signaling pathway-related genes. At 24 hpi, no genes were differentially upregulated in response to Cg. **(D)** DEGs associated with transcription factors (TFs) during resistant interactions with Ca and Cg at 24 and 48 hpi when compared with mock at 0 hpi.

Sixteen defense-related genes were significantly upregulated at 24 hpi (absolute log_2_ fold change >3) in resistant interactions (Figure 5A; Supplementary Table S3). Importantly, seven chitinase (strawberry CLC_DN25_c1204_g1204, strawberry CLC_DN25_c23078_g23078, strawberry CLC_DN25_c25457_g25457, strawberry CLC_DN25_c12430_g12430, strawberry CLC_DN25_c26758_g26758, strawberry CLC_DN25_c41604_g41604, and strawberry CLC_DN25_c7218_g7218), three peroxidase (strawberry CLC_DN25_c39268_g39268, strawberry CLC_DN25_c29676_g29676, and strawberry CLC_DN25_c53035_g53035), two major MLP-like protein 44 (strawberry CLC_DN25_c8109_g8109 and strawberry CLC_DN25_c4034_g4034), one of each pathogenesis-related protein 10 (strawberry CLC_DN25_c29038_g29038), drug resistance protein (strawberry CLC_DN25_c4058_g4058), cinnamate β-D glycosyltransferase (strawberry CLC_DN25_c5236_g5236), and mitochondrial phosphate carrier protein 2 (strawberry CLC_DN25_c216_g216) (Figure 5A; Supplementary Table S3) were upregulated at 24 hpi against Ca. Likewise, the chitinase gene (strawberry CLC_DN25_c1204_g1204) was upregulated at 24 and 48 hpi. Only one chitinase IV gene (strawberry CLC_DN25_c12430_g12430) was upregulated in the resistant interaction at 48 hpi with Cg whereas MLP 44 (strawberry CLC_DN25_c4034_g4034) was upregulated in resistant interactions at 24 and 48 hpi, respectively, by both Ca and Cg (Figure 5A).

### Activation of Genes Related to Immune Receptors (Proteins) and Signal Transduction in Resistant Interactions

The 20 DEGs related to immune receptors and signal transduction were significantly unregulated in the resistant interaction for at least one timepoint. Among them, we found 11 receptor kinases or immune proteins, including LRR receptor-like serine/threonine-protein kinase, protein kinases receptor kinase 1, serine/threonine-protein kinase, and mitogen-activated protein kinase kinase kinase (MAPKKK). One gene, LRR receptor-like serine/threonine-protein kinase (strawberry CLC_DN25_c41090_g41090), was significantly upregulated in resistant interactions at 24 and 48 hpi with Ca, and at 48 hpi with Cg (Figure 5B; Supplementary Table S3). Although two LRR receptor-like serine/threonine-protein kinase genes (strawberry CLC_DN25_c12968_g12968 and strawberry CLC_DN25_c41090_g41090) were upregulated in the resistant interaction at 48 hpi, no gene was upregulated at 24 hpi against Cg. Five calcium-dependent proteins and calcium-dependent protein kinase 2 (strawberry CLC_DN25_c18777_g18777, strawberry CLC_DN25_c19695_g19695, strawberry CLC_DN25_c8902_g8902, strawberry CLC_DN25_c20477_g20477, and strawberry CLC_DN25_c14217_g14217) were also upregulated at 24 hpi with Ca. In addition, ATP-dependent-phosphofructokinase 6 (strawberry CLC_DN25_c13606_g13606) was also upregulated in the resistant interaction at 24 hpi with Ca. Interestingly, one gene involved in the auxin signaling pathway (strawberry CLC_DN25_c5560_g5560) was exclusively upregulated in resistant interactions with Ca at 24 and 48 hpi. At the same time, this activity was also visualized at 48 hpi with Cg (Figure 5C). Although four genes related to lipid metabolism and signaling were upregulated in resistant interactions at both 24 and 48 hpi, more genes were upregulated at 24 hpi with Ca (Figure 5C; Supplementary Table S3).

### Influence of TFs on the Cellular Responses in Resistant Interactions

Sixteen TFs such as NAC, MADS, MYB, WRKY, RNase III protein (a global regulator of gene expression), and auxin response factors were upregulated in resistant interactions with Ca at 24 hpi; however, these TFs were detected with little or no expression at 48 hpi with Ca (Figure 5D; Supplementary Table S3). Conversely, no TF was upregulated at 24 hpi but three TF auxin response factors, MADS, and hypothetical protein T07_921 with unknown functions were upregulated in the resistant interaction to Cg at 48 hpi. Auxin response factors (strawberry CLC_DN25_c5560_g5560) were upregulated in resistant interactions with both Ca and Cg, supporting our hypothesis that these TFs can play an important role in the early plant defense.

### Modulation of Phenylpropanoid Pathway (PPP)–Related Genes in Susceptible Interactions

**FIGURE 6:**
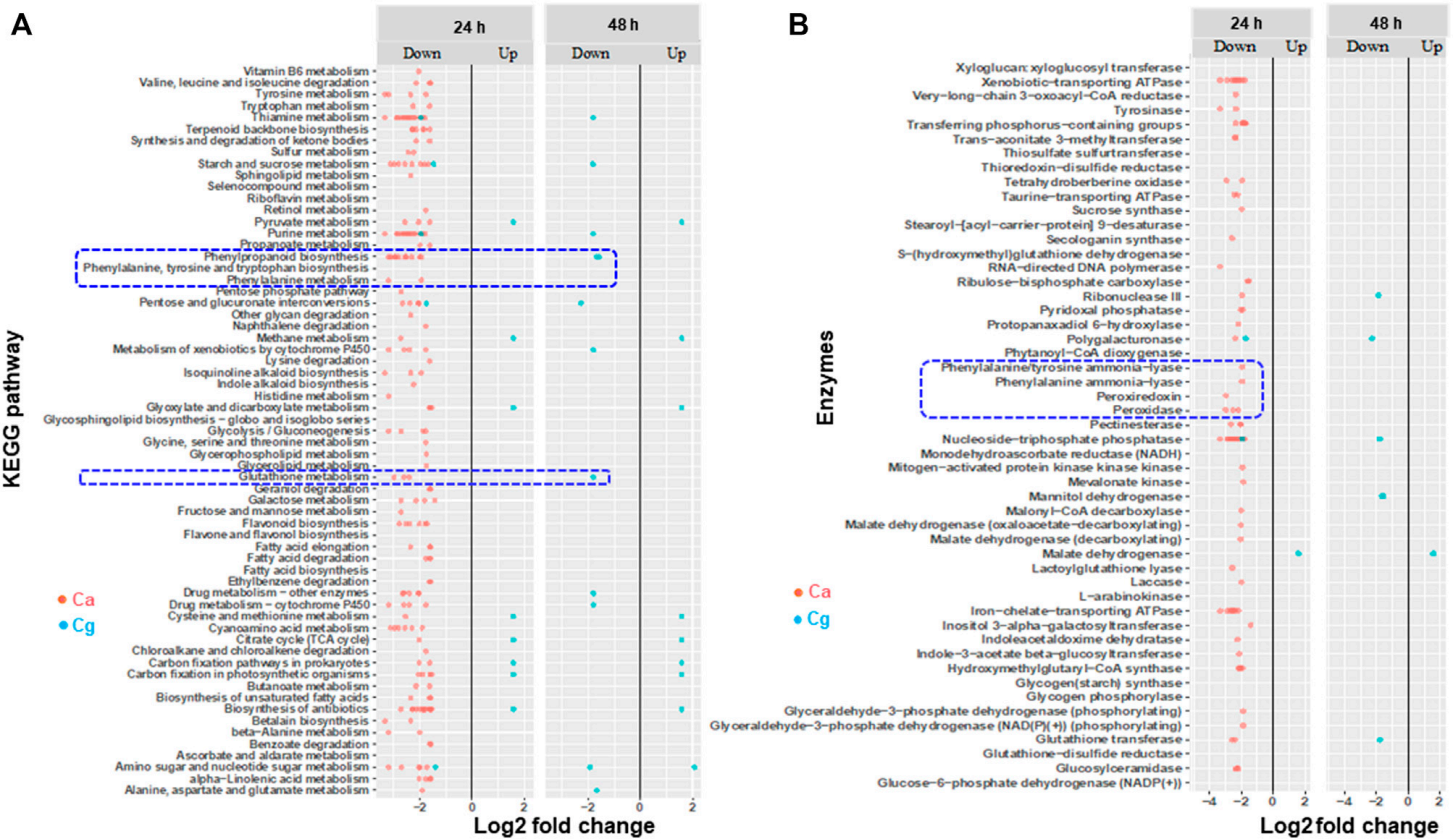
Enriched Kyoto Encyclopedia of Genes and Genomes (KEGG) pathways and genes encoding enzymes that are differentially expressed during susceptible interactions with *Colletotrichum acutatum* (Ca) and *C. gloeosporioides* (Cg). Comparisons were made between susceptible interactions and resistant interactions at 0, 24, and 48 h post-inoculation (hpi). The absolute value of log2 fold change (FC) ≥ |1| and false discovery rate *p* < 0.05 were calculated. **(A)** KEGG pathways. **(B)** Enzymes. Absolute fold change is plotted on the *x*-axis and KEGG and enzymes are plotted on the *y*-axis. The brightness of red color as indicated by the gene expression value in each rectangular is from low to high.

We found several metabolic processes, pathways, and enzymes related to PPP that were downregulated in susceptible interactions at 24 and 48 hpi depending on the pathogen. For example, more biosynthesis pathways and metabolic processes were exclusively downregulated in the susceptible interaction with Ca at 24 hpi than at 48 hpi (Figure 6A), but the expression of these pathways was not observed with Cg. Notably, both PPP and glutathione metabolism were downregulated in the susceptible interaction with Ca at 24 hpi and with Cg at 48 hpi. Two enzymes, phenylalanine ammonia-lyase (PAL) and phenylalanine tyrosine ammonia-lyase, and other enzymes such as ATPase, peroxidase, peroxiredoxin, glutathione s-transferase (GSH), glutathione disulfide reductase, and laccase were downregulated in susceptible interactions with Ca at 24 hpi and with Cg at 48 hpi (Figure 6B; Supplementary Table S4).

### Validation of Selected DEGs by qPCR

The RNA-seq and gene expression results were validated by qPCR using 10 selected DEGs. The expression patterns of all genes showed the same trend in the transcriptome analysis and the qPCR analysis (Supplementary Figure S5).

### Gene Regulatory Network Analysis

**FIGURE 7:**
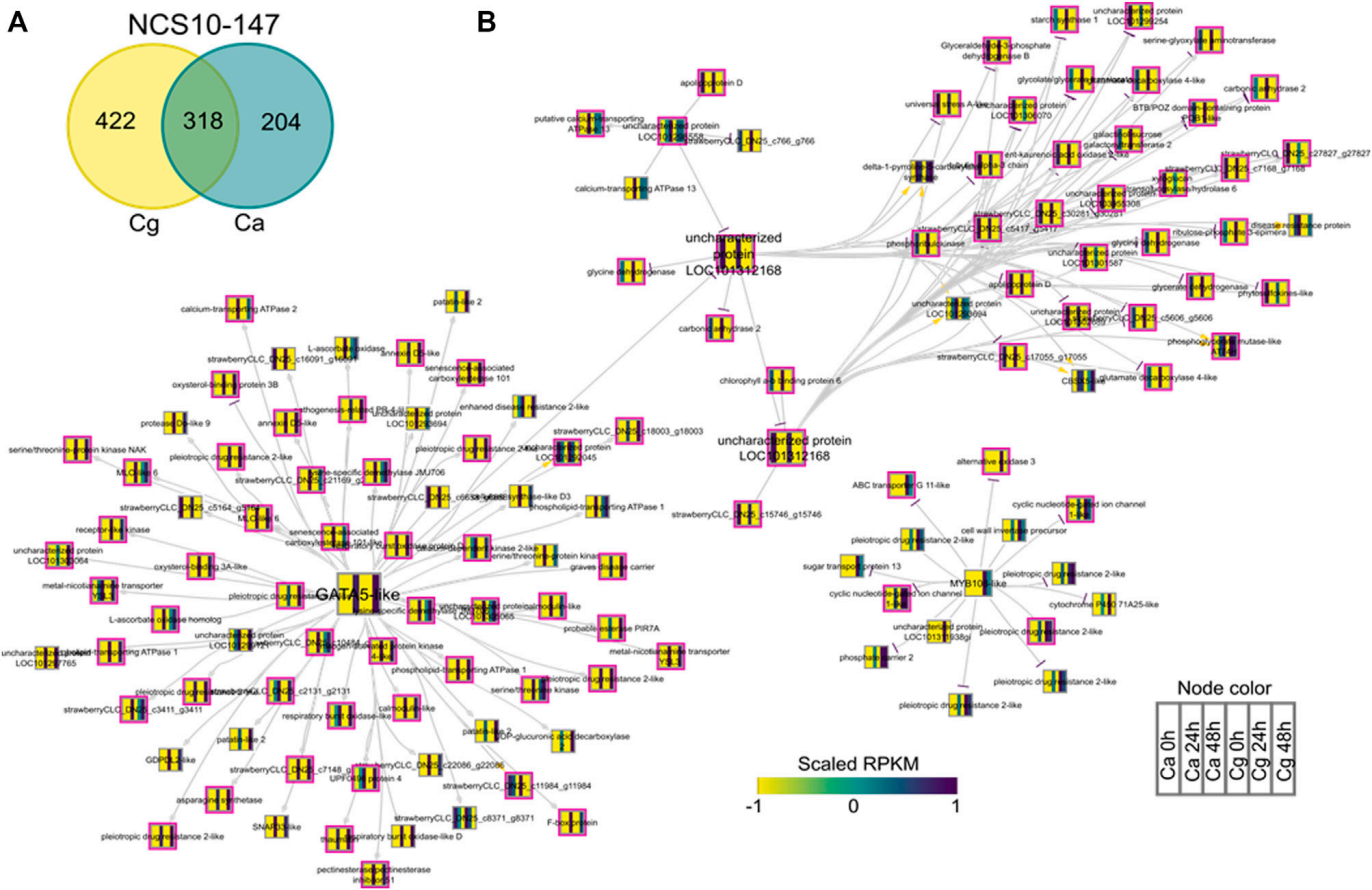
Network analysis of differentially expressed genes (DEGs) during resistant interactions with *Colletotrichum acutatum* (Ca) and *C. gloeosporioides* (Cg). The pairwise comparisons were made at 0–24 h post-inoculation (hpi) and 0–48 hpi using FDR <0.05 and the log2FC ≥ |1| as selection criteria. **(A)** Venn diagram of the DEGs during resistant interactions with Ca and Cg. The 318 common genes were selected for network analysis. **(B)** Causal relations were identified between differentially expressed transcription factors and DEGs through a regression tree with a random forest approach. Yellow, gray, and purple arrowheads represent upregulated, undetermined, and downregulated patterns, respectively. The nodes are colored according to the expression pattern of the gene upon Ca and Cg infection. Pink bordered nodes are also found in the resistant interaction network.

**FIGURE 8:**
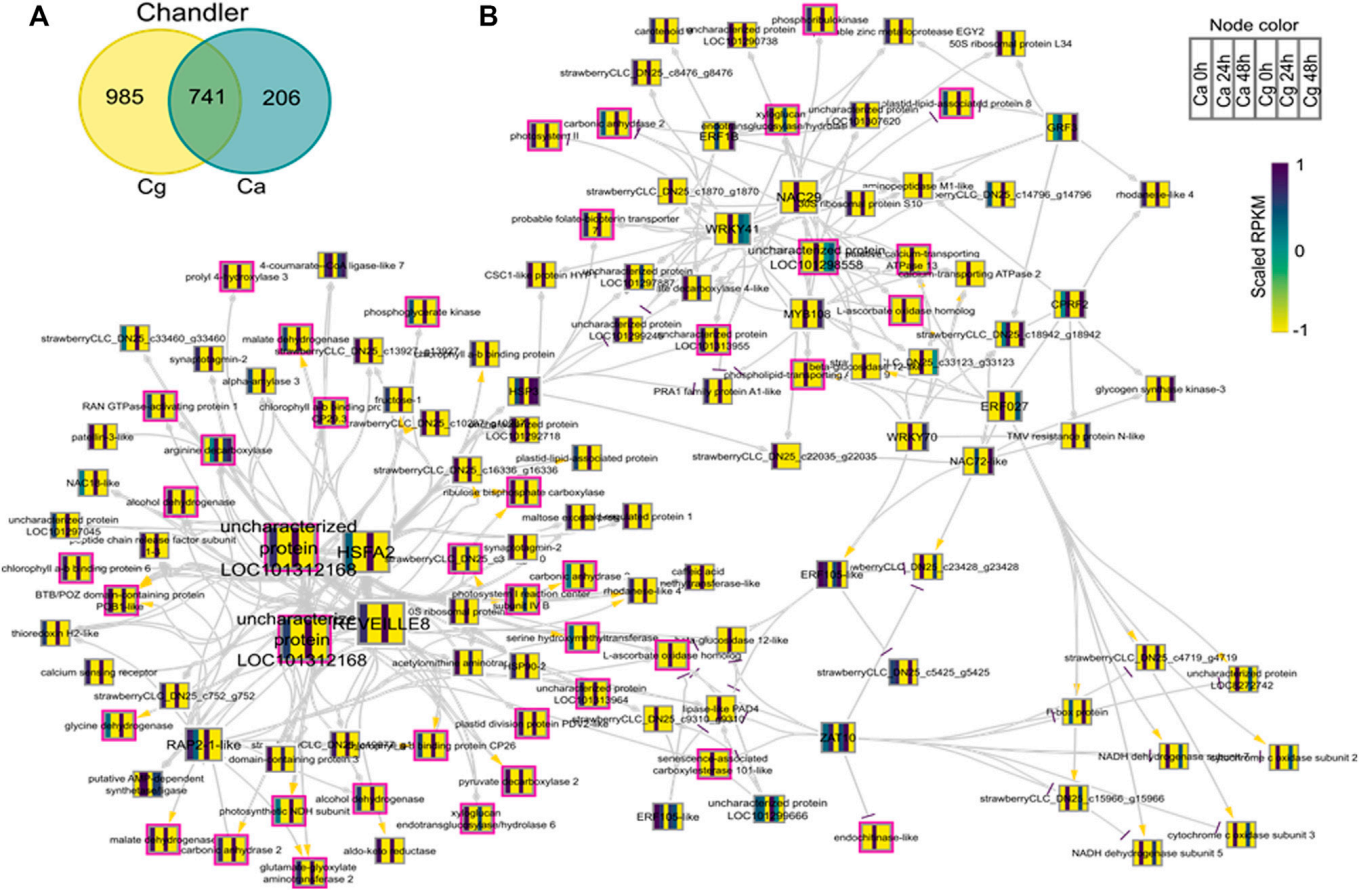
Network analysis of differentially expressed genes (DEGs) during susceptible interactions with *Colletotrichum acutatum* (Ca) and *C. gloeosporioides* (Cg). The pairwise comparison was made at 0–24 h post-inoculation (hpi) and 0–48 hpi using FDR <0.05 and the log2FC ≥ |1| as selection criteria. **(A)** Venn diagram of the DEGs during susceptible interactions. The 741 common genes were selected for network analysis. **(B)** Causal relations were identified through network inference with a regression tree with a random forest approach. Yellow, gray, and purple arrowheads represent upregulated, undetermined, and downregulated patterns, respectively. The nodes are colored according to the expression pattern of the gene upon Ca and Cg infection. Pink bordered nodes are also found in the resistant interaction network.

To identify key regulators associated with target genes revealing distinct biological functions and pathways common to both Ca and Cg, a GRN inference approach was used for both resistant and susceptible interactions. We hypothesized that a similar transcriptional response to both Ca and Cg would suggest that the same genes control host resistance. With increasing the mapping stringencies, the number of TFs mapped to both all transcriptomes’ data set assembly and only the DEG subset between 0 and 24 or 0 and 48 hpi decreased (Table 1). We also identified the DEGs that were common between Ca and Cg concurrent for time. In total, 318 and 741 DEGs were overlapping for the two pathogens during observed resistant responses and susceptible responses, respectively (Figures 7, 8). Four hub regulators, i.e., genes with an increased number of outgoing edges, three uncharacterized proteins, GATA5-like, and MYB10-like have been identified in the resistant interactions (Figures 7A,B). The three major uncharacterized TFs were also responsive to Ca- and Cg-inoculated treatment in susceptible interactions and predicted as a major regulating TF in the susceptible GRN (Figures 8A,B). In both GRNs, the expression of these three TFs reduced after pathogen infection, a dynamic pattern also observed in their predicted downstream targets. On the other hand, GATA5-like and MYB10-like and their downstream targets were activated upon both pathogen infections.

**TABLE 1:** Mapping the transcription factors (TFs) to all contigs in the transcriptome assembly resulted in a different number of TFs mapped or not mapped to the reference transcriptome sequences. The filtering stringencies increase from left to right.

Type of analysis	No filter	MPI 50 MTC 80^b^	MPI 50 MTC 95	MPI 70 MTC 95	MPI 95 MTC 95
TF to all transcripts					
TFs mapped	959 (77%)^a^	736 (59%)	676 (65%)	557 (45%)	355 (24%)
TFs did not map	291 (23%)	514 (41%)	574 (45%)	693 (55%)	895 (76%)
TFs to only DEGs^c^					
TFs mapped	853 (68%)	361 (29%)	496 (40%)	574 (44%)	216 (17%)
TFs did not map	397 (32%)	889 (71%)	754 (60%)	703 (56%)	1,034 (83%)

a Numbers in parentheses correspond to the percentage of total (1,250) strawberry TFs.

b MPI = minimum percent identity, MTC = minimum trimmed coverage.

c The differentially expressed genes (DEGs) were between 0-h timepoint and 24- or 48-h timepoints. There are 75,790 DEGs out of the entire dataset of 77,883 (Additional file 1: Table S1).

## DISCUSSION

Anthracnose caused by Ca and Cg is the most devastating disease in cultivated strawberry (Dickman 2000; Prusky et al., 2000; Peres et al., 2005). We compared transcriptional profiles of strawberry–two hemibiotrophic *Colletotrichum* spp. interactions to identify upregulated and downregulated genes associated with rate-reducing resistance to Ca and Cg at the early infection stages. We found that most genes were significantly upregulated at 24 hpi with Ca, and three candidate genes MLP like protein 44, LRR receptor-like serine/threonine-protein kinase, and auxin-associated transcription factors were associated with resistance to both Ca and Cg. The GRN analysis revealed GATA5-like and MYB10-like TFs as key regulators of the target genes that are involved in downstream defense pathways. To the best of our knowledge, this is the first comparative transcriptome analysis conducted to investigate the early defense responses to both Ca and Cg in a strawberry.

Breeding for anthracnose resistance in strawberries has been primarily focused on identifying new sources of resistance in germplasm collections and breeding selections (Gupton and Smith, 1991; Ballington et al., 2002; Whitaker et al., 2012; Osorio et al., 2014; Anciro et al., 2018). Importantly, current plant breeding and genomic-assisted research are limited due to the octoploid (2*n* = 8*x =*  56) nature of commercial strawberries (Edger et al., 2019). As such, transformation and gene insertion into multiple haplotypes are more difficult and expensive compared with diploid (2*n* = 2*x* = 14) counterparts (Shulaev et al., 2011). As a result, breeding strawberries for resistance to Ca and Cg is challenging due to a lack of genetic information, complex genome, suitable breeding methods, and difficulties encountered in obtaining progeny with the desired traits (Lerceteau-Köhler et al., 2012; Whitaker et al., 2012; Anciro et al., 2018; Edger et al., 2019). In this study, we assessed the phenotypic components of resistance to two hemibiotrophic pathogens in strawberries. Our analysis revealed that more fungal fruitifications or acervuli and higher fungal biomass were detected at 48 hpi than at 24 hpi in the susceptible genotype, which is an indicator of host susceptibility to the hemibiotrophic Ca and Cg. However, low sporulating leaf areas developed in the resistant genotype NCS 10-147 at 0, 24, and 48 hpi are attributed to rate-reducing resistance to Ca and Cg. Consistent with our previous findings (Jacobs et al., 2020), fungal development in the resistant genotype was restricted, preventing further pathogen invasion. In addition, a resistance response that limited the multiplication of the pathogens in the resistant interactions could be associated with the expression of peroxidases and PR and the production of reactive oxygen species (ROS) (Glazebrook, 2005; Dodds and Rathjen, 2010). As a result, very few DEGs were identified for the resistance interactions, thus affecting fungal penetration. Our hypothesis of rate-reducing resistance to Ca and Cg was further supported by the observation that the expression of peroxidases and PR proteins in the resistant genotype NCS 10-147, which are known to be associated with the production of reactive oxygen species (ROS) (Wojtaszek, 1997). Overall, the bioassay approach used in this study provided an insight into identifying a novel source of resistance to both Ca and Cg. The detailed genotyping by sequencing of 280 biparental populations developed from the NCS 10-147 (male parent) × NCS 10-080 (female parent) and association mapping of QDR to Ca and Cg is in progress (Jiménez 2020). This study is designed to confirm our hypothesis of genetic inheritance whether resistance to Ca and Cg in the resistant genotype NCS 10-147 is controlled by either only one gene or multiple genes.

An intriguing observation was that the resistant genotype exhibited the strongest transcriptional responses to Ca and Cg and initiated a defense response at the early stages of infection. Our data provide evidence that defense-related genes (e.g., chitinases I to IV, peroxidases, MLP like proteins, PR protein 10) are upregulated at the early defense response (24 hpi) that were later (48 hpi) unexpectedly downregulated (only two defense-related genes upregulated) after the establishment of the biotrophic phase (Doehlemann et al., 2008; Rudd et al., 2015; Ma et al., 2018). Remarkably, disease resistance genes such as LRR and RLKs, and serine/threonine-protein kinases were upregulated in resistant interactions (Afzal et al., 2008; Pitzschke et al., 2009; Tena et al., 2011; Saijo et al., 2018). Pathogen recognition by surface receptors activates host defense response to pathogen attack and confers basal plant resistance (Asai et al., 2002; Goff and Ramonell, 2007; Tena et al., 2011). Both plants and animals share common innate immune pathways such as PTI and ETI (Ngou et al., 2021), which use specific disease resistance proteins (e.g., PT1, PTI4, and RPS2), leading to HR and PCD (Ausubel, 2005; Andolfo and Ercolano, 2015). Several reports have shown that a single *R* gene or locus confers resistance to multiple pathogens. In *Arabidopsis*, the *RCH1* locus encodes two NBS-LRR proteins that confer resistance to *C. higginsianum* (Narusaka et al., 2004; Narusaka et al., 2009). In addition, both R proteins involved in resistance to bacterial pathogens: RRS1 (*resistance to Ralstonia solanacearum 1*) confers resistance to *R. solanacearum* strain Rs1000, and RPS4 conveys resistance to *Pseudomonas syringae* pv. *tomato* strain DC3000 expressing *avrRps4* (*Pst*—avrRps4) (Narusaka et al., 2009). The *Xa21* gene, which was the first cloned RLK gene in rice, exhibited resistance to multiple strains of *Xanthomonas oryzae* pv. *oryzae* (Song et al., 1995). Subsequently, heterologous expression of *Xa21* in dicots and monocot plants (Tripathi et al., 2014; Holton et al., 2015; Omar et al., 2018) was found to be resistant to multiple bacterial plant pathogens. Likewise, tomato-resistant plants carrying the *Pto* (serine-threonine protein kinase) gene can play a critical role in the signal transduction pathway to *P*. *syringae* pv. tomato (Martin et al., 1993). Furthermore, the activation of the MAPK cascade-mediated pathway also plays an important role in both PTI and ETI signaling (Asai et al., 2002; Pitzschke et al., 2009; Bigeard et al., 2015; Saijo et al., 2018). We found that NBS-LRR, LRR, or RLK proteins appear to be important for mounting host defense and confer resistance to two hemibiotrophic pathogens. Future efforts to apply an RNA interference (RNAi) or gene deletion validation may offer a new tool to probe the role of multiple genes involved in the strawberry defense pathway against two *Colletotrichum* spp.

Both phenylpropanoid and flavonoid pathways play a central role in plant defense (Dixon et al., 2002). In general, phenylpropanoid compounds such as flavonoids, isoflavonoids, anthocyanins, plant hormones, phytoalexins, and lignin can emerge during the response of plants to pathogen attacks. Furthermore, the set of genes involved in the phenylpropanoid pathway (Figure 6) have some mechanisms of defense, including the role from lignification, utilization of secondary metabolites to elicitors, and defense singling regulations (Dixon et al., 2002). Our results indicated that the key genes encoding enzymes (e.g., PAL, CHS) associated with phenylpropanoid pathway and glutathione metabolism were significantly downregulated at 24 hpi. We hypothesize that the downregulation of key genes involved in phenylpropanoid biosynthesis may be due to the delivery of pathogen effector proteins that suppress the expression of the host genes and may assist in pathogen proliferation and pathogenesis at the early stage of infection (Kandel et al., 2020). Our results are consistent with those of previous studies, which also found marked differences in magnitude and temporal expression patterns in metabolism pathways and host defense genes strongly downregulated in different pathosystems (Dowd et al., 2004; Moy et al., 2004; Shimizu et al., 2007; Adhikari et al., 2009; Wang et al., 2017).

The most outstanding discovery of this study was identifying candidate TF genes encoding auxin response factors, NAC, MYB, and WRKY that were differentially upregulated in resistant interactions. These TFs are likely positive regulators associated with host defense and downstream immune responses in strawberries during the asymptomatic phase of infection. We also found that the WRKY75-like gene (CLC_DN25_c39240_g39240) was upregulated in resistant interactions. Several studies have demonstrated that MAPK signaling cascades and WRKY are important in host defense against bacterial and fungal pathogens (Eulgem and Somssich, 2007; Ma et al., 2018). Overexpression of the *Fragaria* FaWRKY1, a homolog of WRKY75, was associated with resistance to *P. syringae* pv. *tomato* (Encinas-Villarejo et al., 2009), and the WRKY33 gene was activated in response to Ca in strawberry (Wang et al., 2017) and *Sclerotinia sclerotiorum* in *Brassica* (Wang et al., 2014). Many NAC TFs also play an important role in plant immunity against plant pathogens (Yuan et al., 2019). Although large plant-specific NAC TFs have been identified in many plant species, only a few NAC members have been described for their biological functions (Fang et al., 2008). In wheat, TF TaNAC8 was found to protect plants against the stripe rust pathogen (Xia et al., 2010), whereas ZmNAC41 and ZmNAC100 genes are activated by JA and SA pathways, respectively, and confer resistance to *C. graminicola* in maize (Voitsik et al., 2013). Our data support the hypothesis that NAC proteins such as NAC2, NAC90, and MYB108 may play an important role in plant defense by acting as positive regulators and modulators of resistance to Ca and Cg.

Phytohormones such as auxin (indole-3-acetic acid, IAA), ethylene (ET), jasmonic acid (JA), and salicylic acid (SA) can activate defense responses individually or interact antagonistically or synergistically in several pathosystems (Bari and Jones, 2009; Yang et al., 2019). Plant immunity to biotrophic pathogens is dependent on SA, whereas defense responses to any necrotrophic pathogens act through JA-, ET-, and ROS-dependent pathways (Thomma et al., 1998; Tierens et al., 2002; Glazebrook, 2005; Yang et al., 2019). Our data indicated that the JA-mediated signaling pathway (e.g., strawberry CLC-DN25_c17975_g17975) was upregulated in resistant interaction. Direct evidence to support a role for SA and JA in disease resistance is based on the observation that *Arabidopsis*-resistant plants infected with a model hemibiotrophic fungus, *C. higginsianum*, revealed the induction of some SA- and JA-inducible genes at the early-stage defense responses (Narusaka et al., 2004). The contribution of SA and JA to basal resistance of *Arabidopsis* to the biotrophic clubroot pathogen, *Plasmodiophora brassicae*, showed two different hormonal responses induced in response to the same isolate of *P. brassicae* in different *Arabidopsis* accessions, and synergistic effects between SA and JA pathways have been reported (Lemarié et al., 2015). We found that the auxin response factors (ARFs) (e.g., strawberry CLC-DN25_c5560_g5560) are upregulated and may enhance resistance to both Ca and Cg. The auxin signaling pathway was found to be regulated by the ARF genes and activated plant defenses against several plant pathogens (Dowd et al., 2004; Guilfoyle and Hagen, 2007; Kunkel and Harper, 2018). Further investigation is necessary to confirm whether or not the auxin signaling pathway was involved in disease resistance to Ca and Cg. The activation of defense mechanisms is regulated by TFs (Chen and Ronald, 2011). To gain insights into the potential gene regulators, we further visualized a GRN network of all DEGs and all differentially regulated predicted TFs for the susceptible and resistant genotypes. We then looked for TFs that have the most connections to the downstream DEGs. One significant discovery of our network inference identified GATA5-like and MYB10-like, with the most connections, which are excellent candidates to confer resistance to both hemibiotrophic *Colletotrichum* spp. In line with our prediction of transcriptional regulators, the previously identified novel GATA-type zinc finger domain and a positive regulator of MYB were activated by abiotic stimuli such as wounding, and elicitor treatment of cells was associated with a protein kinase phosphorylation (Sugimoto et al., 2003). A recent study suggests that TF MYB10 is the candidate gene responsible for controlling fruit color in strawberries (Castillejo et al., 2020). Our data support the hypothesis that these TFs could control early transcription reprogramming and act as promising key regulators for the expression of the early defense genes for resistance to both Ca and Cg. This analysis provides a platform for validation of candidate TFs to investigate their potential contributions to host defense to both pathogens.

**FIGURE 9:**
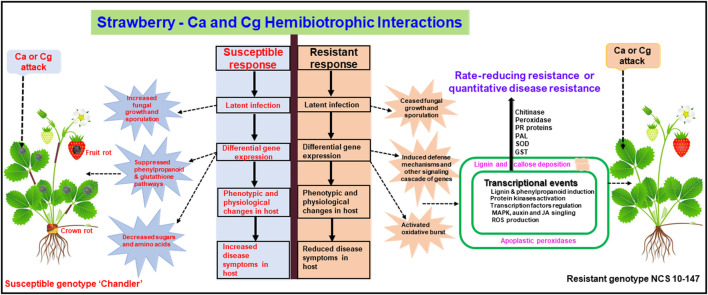
A conceptual model showing upregulated and downregulated genes in resistant interactions (resistant genotype NCS 10-147) and susceptible interactions (susceptible genotype ‘Chandler’) with *Colletotrichum acutatum* (Ca) and *C. gloeosporioides* (Cg). Differentially expressed key genes were upregulated or downregulated at 24 and 48 h post-inoculation (hpi).

In conclusion, we identified the early defense genes in resistant and susceptible interactions in response to two hemibiotrophic *Colletotrichum* spp. (Figure 9). In addition, GRN analysis revealed key two regulators, GATA5-like and MTB10-like, and their target genes in the resistant interactions upon pathogen attacks. Conventional and molecular genetic approaches identified molecular markers linked to *FaRCa1* and *FaRCg1* genes conferring resistance to Ca and Cg, respectively, during the necrotrophic infection in cultivated strawberries (Smith and Spiers 1982; Anciro et al., 2018; Salinas et al., 2019). More recently, Chandra et al. (2021) found the early defense response genes (72 hpi) associated with the *FaRCg1*-mediated resistance to *C*. *gloeosporioides*. Three candidate genes, a von Willebrand Factor A domain-containing protein, a subtilisin-like protease, and a TIFY 11A-like protein, were found to be upregulated in the genomic region of *FaRCg1*. Furthermore, sub-genome-specific markers developed for these candidate genes were used to characterize diverse strawberry accessions from the University of Florida and North Carolina State University breeding programs. It appeared that the gene-specific markers linked to *FaRCg1* were absent in the NCSU mapping population developed from the resistant genotype NCS 10-147 (Jiménez et al., 2020; Chandra et al., 2021), indicating that novel resistance gene(s) or QTLs may present in this population. Our study also identified several early plant defenses in the rate-reducing resistant genotype NCS 10-147 and will be useful to investigate the potential role of these genes conferring resistance to two hemibiotrophic Ca and Cg. This will enable us to develop broad-spectrum (Li et al., 2020) and durable resistance (Johnson, 2000) to both pathogens.

## Data Availability

The datasets presented in this study can be found in online repositories. The names of the repository/repositories and accession number(s) can be found in the article/Supplementary Material.

## Footnotes

1
https://digitalinsights.qiagen.com.

2
https://www.ncbi.nlm.nih.gov/nuccore/LUXP00000000.1.

3
https://www.ncbi.nlm.nih.gov/nuccore/AMYD00000000.1.

4
https://github.com/vsbuffalo/blast2cap3/.

5
https://gex.netlify.com/post/deseq2-experimental-design-and-interpretation/.

6
http://bioinformatics.sdstate.edu/idep/.

7
http://www.genome.jp/kegg/

8
http://strawberry-garden.kazusa.or.jp/.

9
https://github.com/rspurney/TuxNet.

## Supplementary Material

Supplementary Figure S1 Experimental design and sample collection procedures. **(A)** Sequential lifestyle development and colonization of strawberry by *Colletotrichum acutatum* (Ca) and *C. gloeosporioides* (Cg) with some modifications from the previous study (Moraes et al., 2013). **(B)** Strawberry resistant genotype NCS 10-147 (resistant interaction) and susceptible genotype ‘Chandler’ (susceptible interaction) were spray-inoculated with 1.0 × 10^6^ conidia/ml of Ca and Cg. Water (mock) spray-inoculated leaves served as controls. Experiments were conducted two times on greenhouse benches and each experiment had three replications. Samples were collected at 0, 24, and 48 h post-inoculation (hpi). RNA was extracted from each sample, and mRNA-seq libraries were made for each timepoint. Libraries were barcoded and sequenced using an Illumina Hi Seq 2500 platform at the Genomic Sciences Laboratory (GSL) at North Carolina State University, Raleigh, NC.

Supplementary Figure S2 Differentially gene expression and predicted common transcription factors were used for network inference in resistant interactions and susceptible interactions at 0, 24, and 48 h post-inoculation (hpi). To identify significantly expressed genes, the absolute value of log2 fold change (FC) ≥ |1| and false discovery rate *p* < 0.05 were calculated.

Supplementary Figure S3 Summary of gene ontology (GO) terms in resistant interactions in each treatment. To identify significantly expressed genes (DEGs), the absolute value of log2 fold change (FC) ≥ |1| and false discovery rate *p* < 0.05 were calculated. **(A)** A set of significantly upregulated genes associated with biological processes in the resistant interactions with *Colletotrichum acutatum* and *C. gloeosporioides* when compared with mock at 0, 24, and 48 h post-inoculation (hpi). **(B)** A set of significantly upregulated genes associated with molecular functions in resistant interactions when compared with mock at each timepoint. **(C)** A set of significantly upregulated genes associated with cellular components in resistant interactions when compared with mock at each timepoint. **(D)** A set of significantly downregulated genes associated with biological processes in resistant interactions when compared with mock at each timepoint. **(E)** A set of significantly downregulated genes associated with molecular functions in resistant interactions when compared with mock at each timepoint. **(F)** A set of significantly downregulated genes associated with cellular components in resistant interactions when compared with mock at each timepoint.

Supplementary Figure S4 Summary of gene ontology (GO) terms in susceptible interactions in each treatment. To identify significantly expressed genes (DEGs), the absolute value of log2 fold change (FC) ≥ |1| and false discovery rate *p* < 0.05 were calculated. **(A)** A set of significantly upregulated genes associated with biological processes in susceptible interactions with *Colletotrichum acutatum* and *C. gloeosporioides* when compared with mock at 0, 24, and 48 h post-inoculation (hpi). **(B)** A set of significantly upregulated genes associated with molecular functions in susceptible interactions when compared with mock at each timepoint. **(C)** A set of significantly upregulated genes associated with cellular components in susceptible interactions when compared with mock at each timepoint. **(D)** A set of significantly downregulated genes associated with biological processes in susceptible interactions when compared with mock at each timepoint. **(E)** A set of significantly downregulated genes associated with molecular functions in susceptible interactions when compared with mock at each timepoint. **(F)** A set of significantly downregulated genes associated with cellular components in susceptible interactions when compared with mock at each timepoint.

Supplementary Figure S5 Validation of 10 upregulated and downregulated genes by quantitative real-time PCR (qPCR) at 24 and 48 h post-inoculation (hpi) in resistant interactions and susceptible interactions. The glyceraldehyde-3-phosphate dehydrogenase (GAPDH) gene was used as an internal control. Three biological replications were used for the qPCR analysis. Expression of genes is shown in both resistant and susceptible interactions and timepoints and error bars show the SD for qPCR data.
