# Supplementary material for: RNA-Seq and Gene Regulatory Network Analyses Uncover Candidate Genes in the Early Defense to Two Hemibiotrophic Colletorichum spp. in Strawberry
Source: Front Genet. 2022 Mar 10;12:805771. doi: 10.3389/fgene.2021.805771 (PMC8960243; doi:10.3389/fgene.2021.805771)
Supplement: Supplementary file 2 [file Table1.docx]

**Supplementary Table S1.** A list of primer sequences designed from the gene expression data and validated with the Brilliant II SYBR Green master mix quantitative real-time polymerase chain (qPCR) assay.

| **Gene category** | **Strawberry sequence** | **Primer sequences^a^**  **(5’ - 3’)** | **Primer sequence length** | **Annealing temperature (°C)** | **GC%** | **Product size**  **(bp)** | **Primer located on chromosome (Octoploid)** |  |
| --- | --- | --- | --- | --- | --- | --- | --- | --- |
| Defense | StrawberryCLC_DN25_c464_g464 |  |  |  |  |  |  |  |
|  |  | F: TGCATAGTCCTAGACTCTCTCC | 22 | 62 | 50 | 94 | 7-1, 7-2, 7-4 |  |
|  |  | R: CAGCTCAAGCATGAAGCAAC | 20 | 62 | 50 |  | 7-1, 7-2, 7-4 |  |
| Signal transduction | StrawberryCLC_DN25_c43985_g43985 |  |  |  |  |  |  |  |
|  |  | F: AGAATTGTATCCTTGCCTTCCA | 22 | 62 | 40.9 | 103 | 3-2, 3-3 |  |
|  |  | R: GGTTTGCGATCATTGTTCTGTC | 22 | 62 | 45.5 |  | 3-2, 3-3, 3-4 |  |
| Transcription factor | StrawberryCLC_DN25_c21246_g21246 |  |  |  |  |  |  |  |
|  |  | F: AATCCCAGCAGGACCAAAG | 19 | 62 | 52.6 | 109 | 3-1, 3-2, 3-4 |  |
|  |  | R: TGCTTGTGAGGATGCAGAG | 19 | 62 | 52.6 |  | 3-1, 3-2, 3-4 |  |
| Lipid | StrawberryCLC_DN25_c475_g475 |  |  |  |  |  |  |  |
|  |  | F: GTGGTGGTGCTGATCTTGTA | 20 | 62 | 50 | 83 | 6-2 |  |
|  |  | R: ATCAAAGGACTCAACCCTAACC | 22 | 62 | 45.5 |  | 6-2, 6-4 |  |
| Signal transduction | StrawberryCLC_DN25_c9644_g9644 |  |  |  |  |  |  |  |
|  |  | F: GAGATGGTCTGCTGGATGTAG | 21 | 62 | 52.4 | 95 | 3-2, 3-4 |  |
|  |  | R: ATGGCCCTCAAGGACTTC | 18 | 61 | 55.6 |  | 3-2, 3-3, 3-4 |  |
| Defense | StrawberryCLC_DN25_c1204_g1204 |  |  |  |  |  |  |  |
|  |  | F: CCATATTGCTGTCTTGAATGCT | 22 | 61 | 40.9 | 107 | 1-1, 1-3, 1-4 |  |
|  |  | R: ACAATTACAACTATGCTCAAGCG | 23 | 62 | 39.1 |  | 1-1, 1-3, 1-4 |  |
| Transcription factor | StrawberryCLC_DN25_c4215_g4215 |  |  |  |  |  |  |  |
|  |  | F: CACAGAAACTCCACAGGGATATAG | 24 | 62 | 45.8 | 94 | 6-2, 6-3 |  |
|  |  | R: AAAGAAGTCTCAGATTCCCATCC | 23 | 62 | 43.5 |  | 6-2, 6-3 |  |
| Transcription factor | StrawberryCLC_DN25_c4058_g4058 |  |  |  |  |  |  |  |
|  |  | F: ACTGTCCAACCCTGTTGATATT | 22 | 62 | 40.9 | 127 | 3-2, 3-3, 3-4 |  |
|  |  | R: TCTGGTGGGTGATGAAATGAG | 21 | 62 | 47.6 |  | 3-2, 3-3, 3-4 |  |
| Signal transduction | StrawberryCLC_DN25_c12968_g12968 |  |  |  |  |  |  |  |
|  |  | F: TCACCACTTAAGTCACCAATCC | 22 | 62 | 45.5 | 99 | 3-1, 3-2, 3-3 |  |
|  |  | R: CTTAGAGATGATAGCGAGTGGTTT | 24 | 62 | 41.7 |  | 3-1, 3-2, 3-3 |  |
| Hormone signaling | StrawberryCLC_DN25_c5560_g5560 |  |  |  |  |  |  |  |
|  |  | R: GACCCTTCTGATTAGAGTCCAAG | 23 | 62 | 47.8 | 125 | 5-1, 5-2, 5-3, 5-4 |  |
|  |  | F: AGCTGTAGCATCTTCTAATTCCA | 23 | 62 | 39.1 |  | 5-1, 5-2, 5-3, 5-4 |  |
|  |  |  |  |  |  |  |  |  |

^a^ F = forward and R = reverse primers are given in the table were used for qPCR assay. Glyceraldehyde-3-phosphate dehydrogenase (*GAPDH*) gene was used as an internal control.
