# Supplementary material for: RNA-Seq and Gene Regulatory Network Analyses Uncover Candidate Genes in the Early Defense to Two Hemibiotrophic Colletorichum spp. in Strawberry
Source: Front Genet. 2022 Mar 10;12:805771. doi: 10.3389/fgene.2021.805771 (PMC8960243; doi:10.3389/fgene.2021.805771)
Supplement: Supplementary file 3 [file Presentation1.PPTX]

## Slide 1
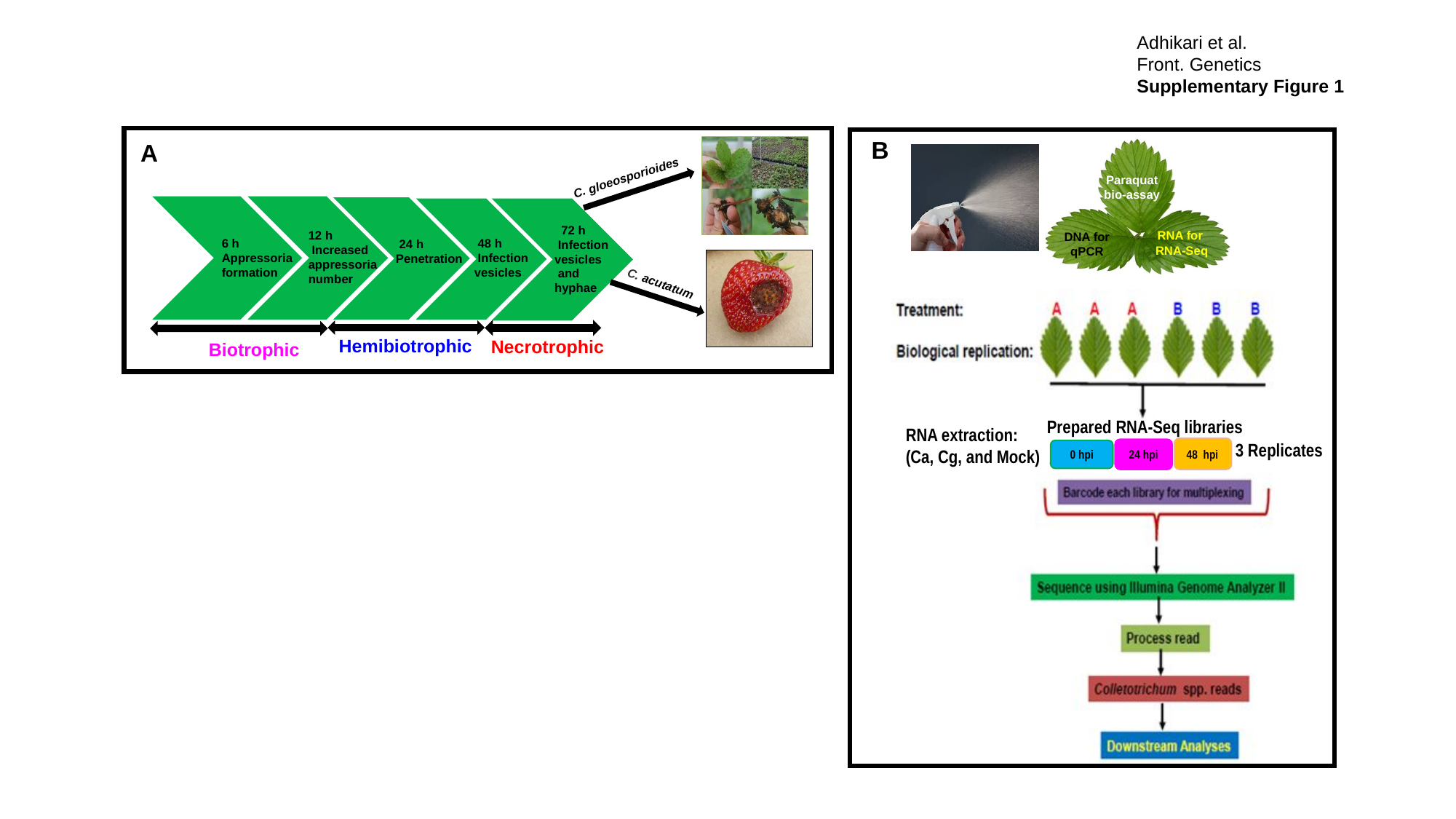

Adhikari et al.
Front. Genetics
Supplementary Figure 1
6 h
Appressoria
formation
12 h
 Increased
appressoria
number
 48 h
 Infection
vesicles
 24 h
 Penetration
 72 h
 Infection vesicles
 and hyphae
Hemibiotrophic
Necrotrophic
Biotrophic
C. gloeosporioides
C. acutatum
A
 Paraquat
bio-assay
B
 Paraquat
bio-assay
RNA for
RNA-Seq
DNA for qPCR
Prepared RNA-Seq libraries
RNA extraction:
(Ca, Cg, and Mock)
3 Replicates
48 hpi
24 hpi
0 hpi
