# Supplementary figures and images for: RNA-Seq and Gene Regulatory Network Analyses Uncover Candidate Genes in the Early Defense to Two Hemibiotrophic Colletorichum spp. in Strawberry
Source: Front Genet. 2022 Mar 10;12:805771. doi: 10.3389/fgene.2021.805771 (PMC8960243; doi:10.3389/fgene.2021.805771)

## Slide 1
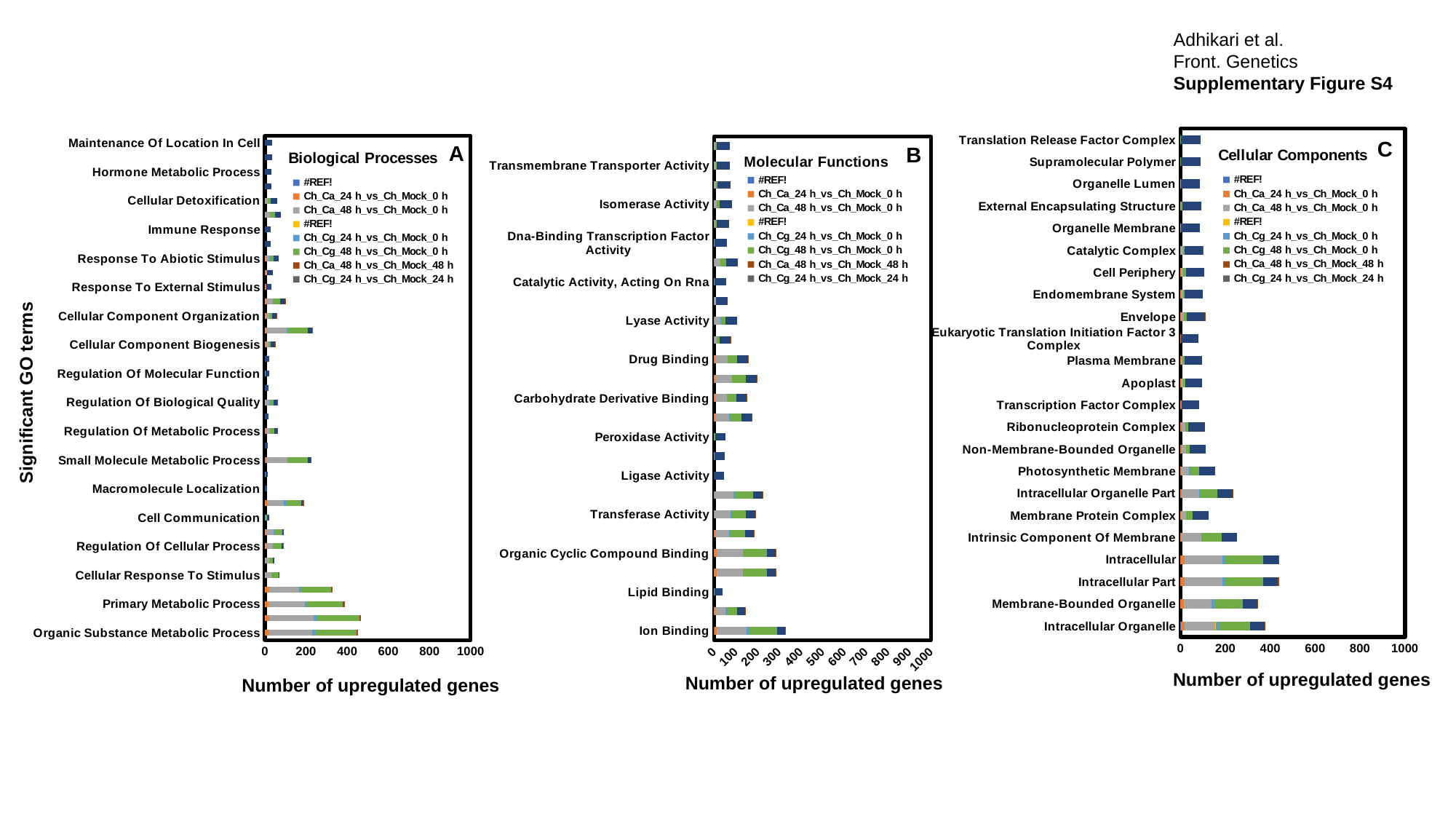

## Slide 2
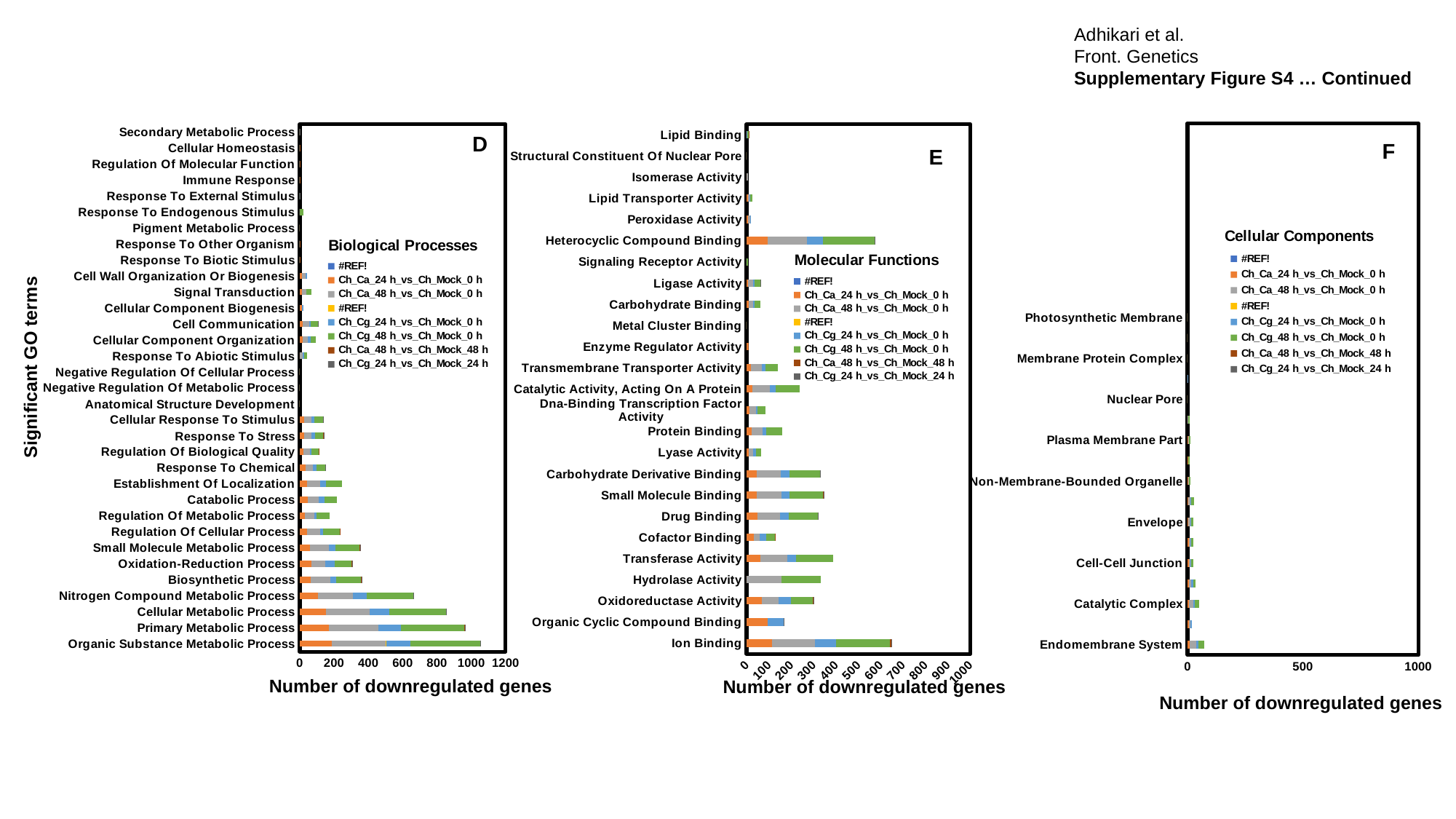

Supplement: Supplementary file 4 [file Presentation4.PPTX]

## Slide 1
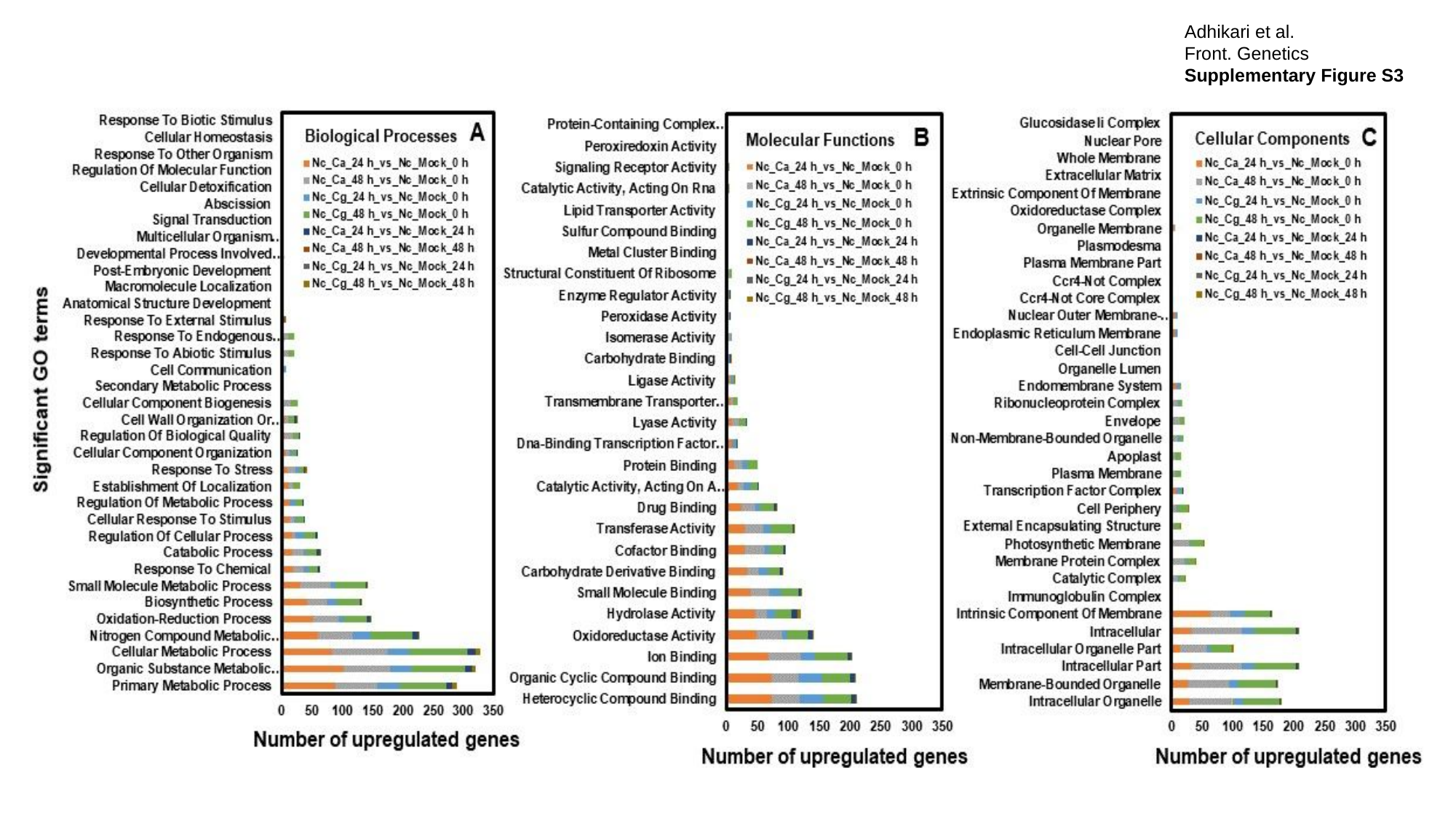

## Slide 2
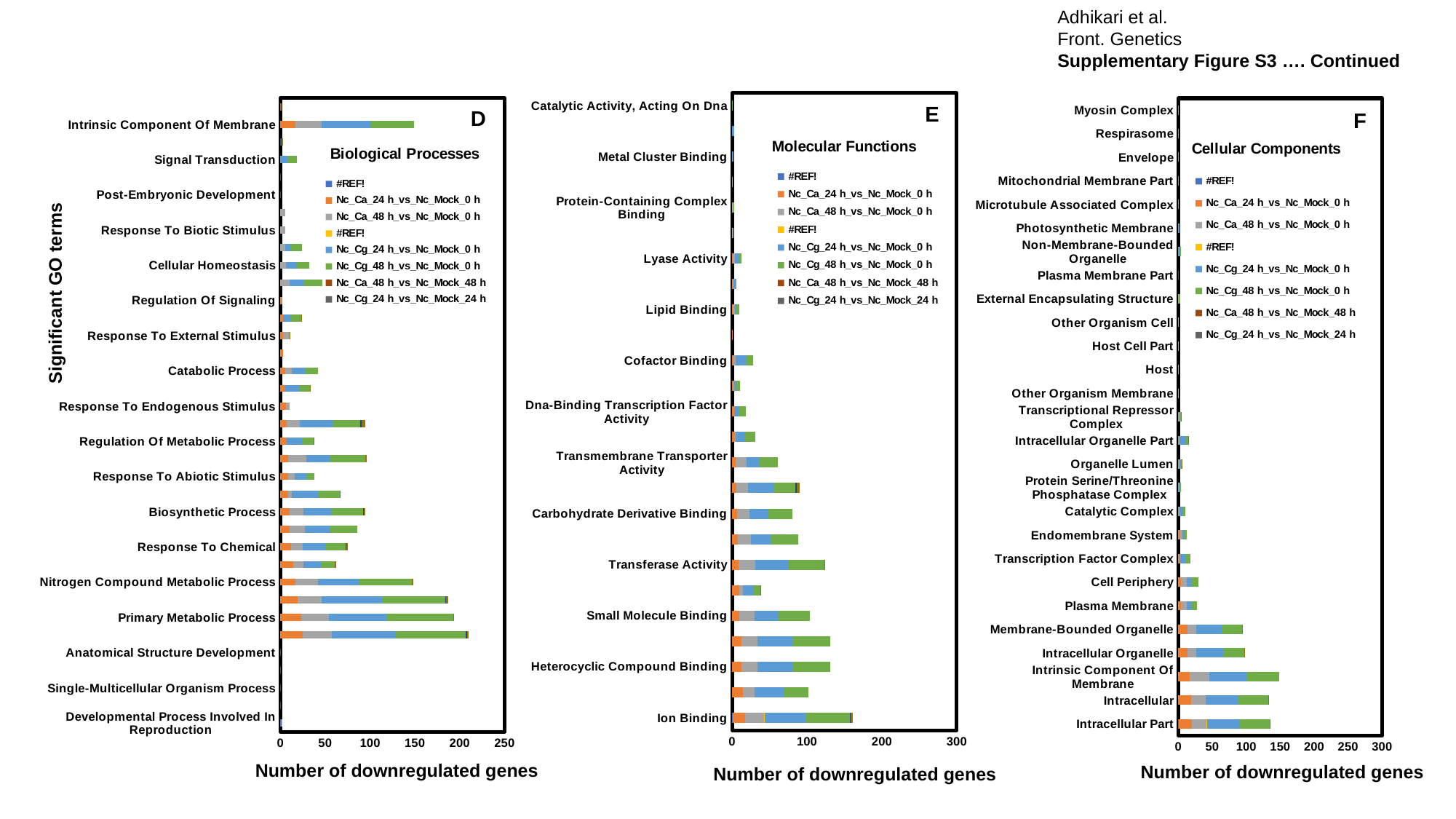

Supplement: Supplementary file 7 [file Presentation3.PPTX]

## Slide 1
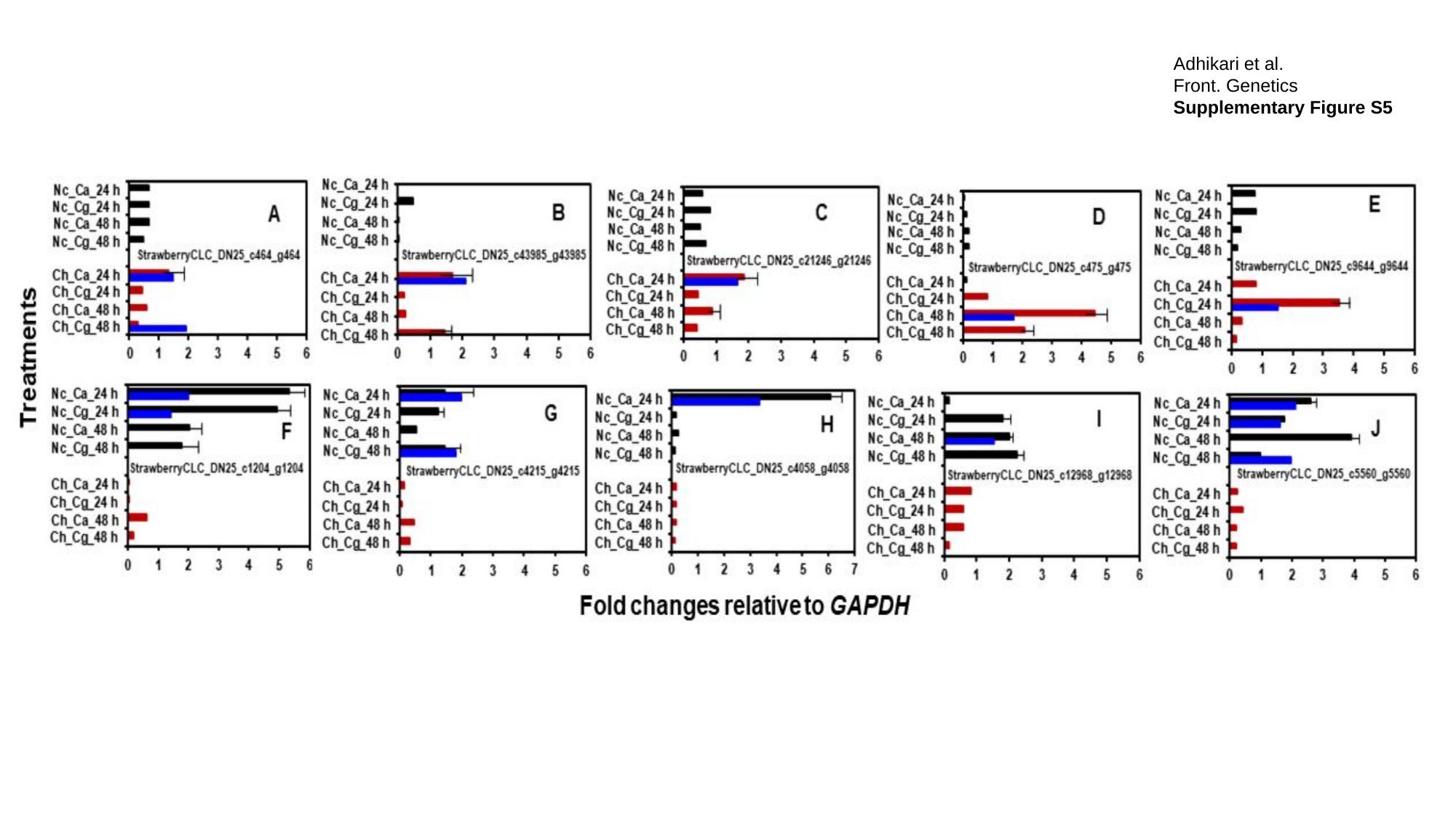

Adhikari et al.
Front. Genetics
Supplementary Figure S5

Supplement: Supplementary file 9 [file Presentation5.PPTX]
