# Supplementary material for: RNA-Seq and Gene Regulatory Network Analyses Uncover Candidate Genes in the Early Defense to Two Hemibiotrophic Colletorichum spp. in Strawberry
Source: Front Genet. 2022 Mar 10;12:805771. doi: 10.3389/fgene.2021.805771 (PMC8960243; doi:10.3389/fgene.2021.805771)
Supplement: Supplementary file 6 [file Table3.docx]

**Supplementary Table S3**. List of defenses, hormone signaling, lipid, signal transduction, and transcription factors associated differentially expressed genes (DEGs) in resistant genotype NCS 10-147 (Nc) versus susceptible genotype ‘Chandler’ (Ch) that were upregulated and downregulated (*P* adjusted < 0.05, Log2fold change >= I1I) genes at 24 and 48 hr. postinoculation (hpi) with *Colletotrichum accutatum* (Ca) and *Colletotrichum gloeosporioides* (Cg). Values with negative signs are downregulated genes. Nonsignificant values are not shown and are listed as blank.

|  |  |  |  |  | **24 hai** | | **48 hai** |
| --- | --- | --- | --- | --- | --- | --- | --- |
| **Sequence** | **GO ID** | **GO Term** | **Gene category** | **Putative gene function** | **Ch-Ca versus** | **Ch-Cg versus** | **Ch-Cg versus** |
|  |  |  |  |  | **Nc-Ca** | **Nc-Cg** | **Nc-Cg** |
| strawberryCLC_DN25_c1204_g1204 | GO:0006032 | Biological process | Defense | chitin catabolic process |  | 1.4 |  |
| strawberryCLC_DN25_c12430_g12430 | GO:0006032 | Biological process | Defense | chitin catabolic process |  |  | 1.93 |
| strawberryCLC_DN25_c15935_g15935 | GO:0006032 | Biological process | Defense | chitin catabolic process |  |  | -2.4 |
| strawberryCLC_DN25_c187_g187 | GO:0006032 | Biological process | Defense | chitin catabolic process |  |  | -2.07 |
| strawberryCLC_DN25_c25457_g25457 | GO:0006032 | Biological process | Defense | chitin catabolic process | 2.03 |  |  |
| strawberryCLC_DN25_c26758_g26758 | GO:0006032 | Biological process | Defense | chitin catabolic process | 2.7 |  |  |
| strawberryCLC_DN25_c41604_g41604 | GO:0006032 | Biological process | Defense | chitin catabolic process | 3.18 |  |  |
| strawberryCLC_DN25_c7218_g7218 | GO:0006032 | Biological process | Defense | chitin catabolic process | 1.73 |  |  |
| strawberryCLC_DN25_c29038_g29038 | GO:0006952 | Biological process | Defense | defense response | 2.71 |  |  |
| strawberryCLC_DN25_c4034_g4034 | GO:0006952 | Biological process | Defense | defense response | 2.3 |  | 1.71 |
| strawberryCLC_DN25_c8109_g8109 | GO:0006952 | Biological process | Defense | defense response | 1.91 |  |  |
| strawberryCLC_DN25_c1139_g1139 | GO:0042742 | Biological process | Defense | defense response to bacterium |  | -1.58 | -1.58 |
| strawberryCLC_DN25_c1204_g1204 | GO:0050832 | Biological process | Defense | defense response to fungus | 1.98 | 1.4 |  |
| strawberryCLC_DN25_c12430_g12430 | GO:0050832 | Biological process | Defense | defense response to fungus |  |  | 1.93 |
| strawberryCLC_DN25_c15935_g15935 | GO:0050832 | Biological process | Defense | defense response to fungus |  |  | -2.4 |
| strawberryCLC_DN25_c29676_g29676 | GO:0006804 | Biological process | Defense | obsolete peroxidase reaction | 2.52 |  |  |
| strawberryCLC_DN25_c39268_g39268 | GO:0006804 | Biological process | Defense | obsolete peroxidase reaction | 2.99 |  |  |
| strawberryCLC_DN25_c53035_g53035 | GO:0006804 | Biological process | Defense | obsolete peroxidase reaction | 2.2 |  |  |
| strawberryCLC_DN25_c1204_g1204 | GO:0008061 | Molecular function | Defense | chitin binding | 1.98 | 1.4 |  |
| strawberryCLC_DN25_c12430_g12430 | GO:0008061 | Molecular function | Defense | chitin binding |  |  | 1.93 |
| strawberryCLC_DN25_c15935_g15935 | GO:0008061 | Molecular function | Defense | chitin binding |  |  | -2.4 |
| strawberryCLC_DN25_c187_g187 | GO:0008061 | Molecular function | Defense | chitin binding |  |  | -2.07 |
| strawberryCLC_DN25_c23078_g23078 | GO:0008061 | Molecular function | Defense | chitin binding | 2.18 |  |  |
| strawberryCLC_DN25_c25457_g25457 | GO:0008061 | Molecular function | Defense | chitin binding | 2.03 |  |  |
| strawberryCLC_DN25_c26758_g26758 | GO:0008061 | Molecular function | Defense | chitin binding | 2.7 |  |  |
| strawberryCLC_DN25_c41604_g41604 | GO:0008061 | Molecular function | Defense | chitin binding | 3.18 |  |  |
| strawberryCLC_DN25_c7218_g7218 | GO:0008061 | Molecular function | Defense | chitin binding | 1.73 |  |  |
| strawberryCLC_DN25_c1204_g1204 | GO:0004568 | Molecular function | Defense | chitinase activity | 1.98 | 1.4 |  |
| strawberryCLC_DN25_c12430_g12430 | GO:0004568 | Molecular function | Defense | chitinase activity |  |  | 1.93 |
| strawberryCLC_DN25_c15935_g15935 | GO:0004568 | Molecular function | Defense | chitinase activity |  |  | -2.4 |
| strawberryCLC_DN25_c187_g187 | GO:0004568 | Molecular function | Defense | chitinase activity |  |  | -2.07 |
| strawberryCLC_DN25_c25457_g25457 | GO:0004568 | Molecular function | Defense | chitinase activity | 2.03 |  |  |
| strawberryCLC_DN25_c26758_g26758 | GO:0004568 | Molecular function | Defense | chitinase activity | 2.7 |  |  |
| strawberryCLC_DN25_c41604_g41604 | GO:0004568 | Molecular function | Defense | chitinase activity | 3.18 |  |  |
| strawberryCLC_DN25_c7218_g7218 | GO:0004568 | Molecular function | Defense | chitinase activity | 1.73 |  |  |
| strawberryCLC_DN25_c29676_g29676 | GO:0004601 | Molecular function | Defense | peroxidase activity | 2.52 |  |  |
| strawberryCLC_DN25_c39268_g39268 | GO:0004601 | Molecular function | Defense | peroxidase activity | 2.99 |  |  |
| strawberryCLC_DN25_c53035_g53035 | GO:0004601 | Molecular function | Defense | peroxidase activity | 2.2 |  |  |
| strawberryCLC_DN25_c5560_g5560 | GO:0009734 | Biological process | Hormone signaling | auxin-activated signaling pathway | 2.11 | 1.61 | 1.97 |
| strawberryCLC_DN25_c17975_g17975 | GO:0009695 | Biological process | Hormone signaling | jasmonic acid biosynthetic process | 2.33 |  |  |
| strawberryCLC_DN25_c5547_g5547 | GO:0046486 | Biological process | Lipid | glycerolipid metabolic process | 1.74 |  |  |
| strawberryCLC_DN25_c13321_g13321 | GO:0016042 | Biological process | Lipid | lipid catabolic process |  |  | -2.11 |
| strawberryCLC_DN25_c9359_g9359 | GO:0016042 | Biological process | Lipid | lipid catabolic process | 2.07 |  |  |
| strawberryCLC_DN25_c105_g105 | GO:0006629 | Biological process | Lipid | lipid metabolic process | 2.08 |  |  |
| strawberryCLC_DN25_c5905_g5905 | GO:0006629 | Biological process | Lipid | lipid metabolic process | 2.26 | 1.89 |  |
| strawberryCLC_DN25_c475_g475 | GO:0006869 | Biological process | Lipid | lipid transport |  |  | -1.69 |
| strawberryCLC_DN25_c5547_g5547 | GO:0008654 | Biological process | Lipid | phospholipid biosynthetic process | 1.74 |  |  |
| strawberryCLC_DN25_c475_g475 | GO:0008289 | Molecular function | Lipid | lipid binding |  |  | -1.69 |
| strawberryCLC_DN25_c14217_g14217 | GO:0070588 | Biological process | Signal transduction | calcium ion transmembrane transport | 1.9 |  |  |
| strawberryCLC_DN25_c43985_g43985 | GO:0007165 | Biological process | Signal transduction | signal transduction | -2.12 |  |  |
| strawberryCLC_DN25_c52886_g52886 | GO:0007165 | Biological process | Signal transduction | signal transduction | 2.19 |  |  |
| strawberryCLC_DN25_c2599_g2599 | GO:0007178 | Biological process | Signal transduction | transmembrane receptor protein serine/threonine kinase signaling pathway | 1.94 |  |  |
| strawberryCLC_DN25_c13606_g13606 | GO:0005945 | Cellular component | Signal transduction | 6-phosphofructokinase complex | 2.72 |  |  |
| strawberryCLC_DN25_c13606_g13606 | GO:0003872 | Molecular function | Signal transduction | 6-phosphofructokinase activity | 2.72 |  |  |
| strawberryCLC_DN25_c18777_g18777 | GO:0005509 | Molecular function | Signal transduction | calcium ion binding | 2.11 |  |  |
| strawberryCLC_DN25_c19695_g19695 | GO:0005509 | Molecular function | Signal transduction | calcium ion binding | 2.35 |  |  |
| strawberryCLC_DN25_c8902_g8902 | GO:0005509 | Molecular function | Signal transduction | calcium ion binding | 1.82 |  |  |
| strawberryCLC_DN25_c14217_g14217 | GO:0005388 | Molecular function | Signal transduction | calcium-transporting ATPase activity | 1.9 |  |  |
| strawberryCLC_DN25_c21981_g21981 | GO:0005516 | Molecular function | Signal transduction | calmodulin binding |  | 1.34 |  |
| strawberryCLC_DN25_c5769_g5769 | GO:0005516 | Molecular function | Signal transduction | calmodulin binding |  | 1.26 |  |
| strawberryCLC_DN25_c19695_g19695 | GO:0004683 | Molecular function | Signal transduction | calmodulin-dependent protein kinase activity | 2.35 |  |  |
| strawberryCLC_DN25_c16592_g16592 | GO:0003677 | Molecular function | Signal transduction | DNA binding | 2.16 |  |  |
| strawberryCLC_DN25_c1675_g1675 | GO:0003677 | Molecular function | Signal transduction | DNA binding | 1.51 |  |  |
| strawberryCLC_DN25_c3290_g3290 | GO:0003677 | Molecular function | Signal transduction | DNA binding |  | 1.63 |  |
| strawberryCLC_DN25_c44188_g44188 | GO:0003677 | Molecular function | Signal transduction | DNA binding | 2.38 |  |  |
| strawberryCLC_DN25_c46523_g46523 | GO:0003677 | Molecular function | Signal transduction | DNA binding | 2.26 | 1.63 |  |
| strawberryCLC_DN25_c5560_g5560 | GO:0003677 | Molecular function | Signal transduction | DNA binding | 2.11 | 1.61 | 1.97 |
| strawberryCLC_DN25_c8143_g8143 | GO:0003677 | Molecular function | Signal transduction | DNA binding | 1.5 |  |  |
| strawberryCLC_DN25_c20477_g20477 | GO:0016301 | Molecular function | Signal transduction | kinase activity | 2.21 |  |  |
| strawberryCLC_DN25_c2599_g2599 | GO:0004709 | Molecular function | Signal transduction | MAP kinase kinase kinase activity | 1.94 |  |  |
| strawberryCLC_DN25_c8876_g8876 | GO:0004496 | Molecular function | Signal transduction | mevalonate kinase activity | 1.89 |  |  |
| strawberryCLC_DN25_c1062_g1062 | GO:0004672 | Molecular function | Signal transduction | protein kinase activity | 1.67 |  |  |
| strawberryCLC_DN25_c12968_g12968 | GO:0004672 | Molecular function | Signal transduction | protein kinase activity |  |  | 1.7 |
| strawberryCLC_DN25_c27263_g27263 | GO:0004672 | Molecular function | Signal transduction | protein kinase activity | 2.02 |  |  |
| strawberryCLC_DN25_c41090_g41090 | GO:0004672 | Molecular function | Signal transduction | protein kinase activity | 1.64 | 1.57 | 1.77 |
| strawberryCLC_DN25_c52886_g52886 | GO:0004672 | Molecular function | Signal transduction | protein kinase activity | 2.19 |  |  |
| strawberryCLC_DN25_c9644_g9644 | GO:0004672 | Molecular function | Signal transduction | protein kinase activity |  | -1.52 |  |
| strawberryCLC_DN25_c18114_g18114 | GO:0004674 | Molecular function | Signal transduction | protein serine/threonine kinase activity | 1.91 |  |  |
| strawberryCLC_DN25_c24203_g24203 | GO:0004674 | Molecular function | Signal transduction | protein serine/threonine kinase activity | 1.81 |  |  |
| strawberryCLC_DN25_c4746_g4746 | GO:0004674 | Molecular function | Signal transduction | protein serine/threonine kinase activity | 1.73 |  |  |
| strawberryCLC_DN25_c9170_g9170 | GO:0004674 | Molecular function | Signal transduction | protein serine/threonine kinase activity | 1.81 |  |  |
| strawberryCLC_DN25_c11696_g11696 | GO:0033883 | Molecular function | Signal transduction | pyridoxal phosphatase activity | 1.91 |  |  |
| strawberryCLC_DN25_c8934_g8934 | GO:0033883 | Molecular function | Signal transduction | pyridoxal phosphatase activity | 2.03 |  |  |
| strawberryCLC_DN25_c464_g464 | GO:0000977 | Molecular function | Signal transduction | RNA polymerase II regulatory region sequence-specific DNA binding | -1.47 |  | -1.91 |
| strawberryCLC_DN25_c21246_g21246 | GO:0043565 | Molecular function | Signal transduction | sequence-specific DNA binding | -1.64 |  |  |
| strawberryCLC_DN25_c22396_g22396 | GO:0043565 | Molecular function | Signal transduction | sequence-specific DNA binding | 2.15 |  |  |
| strawberryCLC_DN25_c26676_g26676 | GO:0043565 | Molecular function | Signal transduction | sequence-specific DNA binding | 2.5 |  |  |
| strawberryCLC_DN25_c39240_g39240 | GO:0043565 | Molecular function | Signal transduction | sequence-specific DNA binding | 2.2 |  |  |
| strawberryCLC_DN25_c52886_g52886 | GO:0005102 | Molecular function | Signal transduction | signaling receptor binding | 2.19 |  |  |
| strawberryCLC_DN25_c39268_g39268 | GO:0034599 | Biological process | Stress | cellular response to oxidative stress | 2.99 |  |  |
| strawberryCLC_DN25_c29676_g29676 | GO:0006979 | Biological process | Stress | response to oxidative stress | 2.52 |  |  |
| strawberryCLC_DN25_c53035_g53035 | GO:0006979 | Biological process | Stress | response to oxidative stress | 2.2 |  |  |
| strawberryCLC_DN25_c1139_g1139 | GO:0009651 | Biological process | Stress | response to salt stress |  | -1.58 | -1.58 |
| strawberryCLC_DN25_c216_g216 | GO:0009651 | Biological process | Stress | response to salt stress | 2.06 |  |  |
| strawberryCLC_DN25_c5236_g5236 | GO:0009636 | Biological process | Stress | response to toxic substance | 1.74 |  |  |
| strawberryCLC_DN25_c464_g464 | GO:0045944 | Biological process | Transcription factor | positive regulation of transcription by RNA polymerase II | -1.47 |  | -1.91 |
| strawberryCLC_DN25_c1675_g1675 | GO:0006355 | Biological process | Transcription factor | regulation of transcription, DNA-templated | 1.51 |  |  |
| strawberryCLC_DN25_c21246_g21246 | GO:0006355 | Biological process | Transcription factor | regulation of transcription, DNA-templated | 1.64 |  |  |
| strawberryCLC_DN25_c22396_g22396 | GO:0006355 | Biological process | Transcription factor | regulation of transcription, DNA-templated | 2.15 |  |  |
| strawberryCLC_DN25_c3290_g3290 | GO:0006355 | Biological process | Transcription factor | regulation of transcription, DNA-templated |  | 1.63 |  |
| strawberryCLC_DN25_c39240_g39240 | GO:0006355 | Biological process | Transcription factor | regulation of transcription, DNA-templated | 2.2 |  |  |
| strawberryCLC_DN25_c464_g464 | GO:0006355 | Biological process | Transcription factor | regulation of transcription, DNA-templated | -1.47 |  | -1.91 |
| strawberryCLC_DN25_c5560_g5560 | GO:0006355 | Biological process | Transcription factor | regulation of transcription, DNA-templated | 2.11 | 1.61 | 1.97 |
| strawberryCLC_DN25_c8143_g8143 | GO:0006355 | Biological process | Transcription factor | regulation of transcription, DNA-templated | 1.5 |  |  |
| strawberryCLC_DN25_c4215_g4215 | GO:0006396 | Biological process | Transcription factor | RNA processing | 1.96 |  | 1.88 |
| strawberryCLC_DN25_c4058_g4058 | GO:0006278 | Biological process | Transcription factor | RNA-dependent DNA biosynthetic process | 3.34 |  |  |
| strawberryCLC_DN25_c21246_g21246 | GO:0005667 | Cellular component | Transcription factor | transcription factor complex | -1.64 |  |  |
| strawberryCLC_DN25_c22396_g22396 | GO:0005667 | Cellular component | Transcription factor | transcription factor complex | 2.15 |  |  |
| strawberryCLC_DN25_c39240_g39240 | GO:0005667 | Cellular component | Transcription factor | transcription factor complex | 2.2 |  |  |
| strawberryCLC_DN25_c464_g464 | GO:0005667 | Cellular component | Transcription factor | transcription factor complex | -1.47 |  | -1.91 |
| strawberryCLC_DN25_c21246_g21246 | GO:0003700 | Molecular function | Transcription factor | DNA-binding transcription factor activity | -1.64 |  |  |
| strawberryCLC_DN25_c22396_g22396 | GO:0003700 | Molecular function | Transcription factor | DNA-binding transcription factor activity | 2.15 |  |  |
| strawberryCLC_DN25_c39240_g39240 | GO:0003700 | Molecular function | Transcription factor | DNA-binding transcription factor activity | 2.2 |  |  |
| strawberryCLC_DN25_c464_g464 | GO:0003700 | Molecular function | Transcription factor | DNA-binding transcription factor activity | -1.47 |  | -1.91 |
| strawberryCLC_DN25_c4058_g4058 | GO:0003964 | Molecular function | Transcription factor | RNA-directed DNA polymerase activity | 3.34 |  |  |
