# Supplementary material for: RNA-Seq and Gene Regulatory Network Analyses Uncover Candidate Genes in the Early Defense to Two Hemibiotrophic Colletorichum spp. in Strawberry
Source: Front Genet. 2022 Mar 10;12:805771. doi: 10.3389/fgene.2021.805771 (PMC8960243; doi:10.3389/fgene.2021.805771)
Supplement: Supplementary file 8 [file Presentation2.PPTX]

## Slide 1
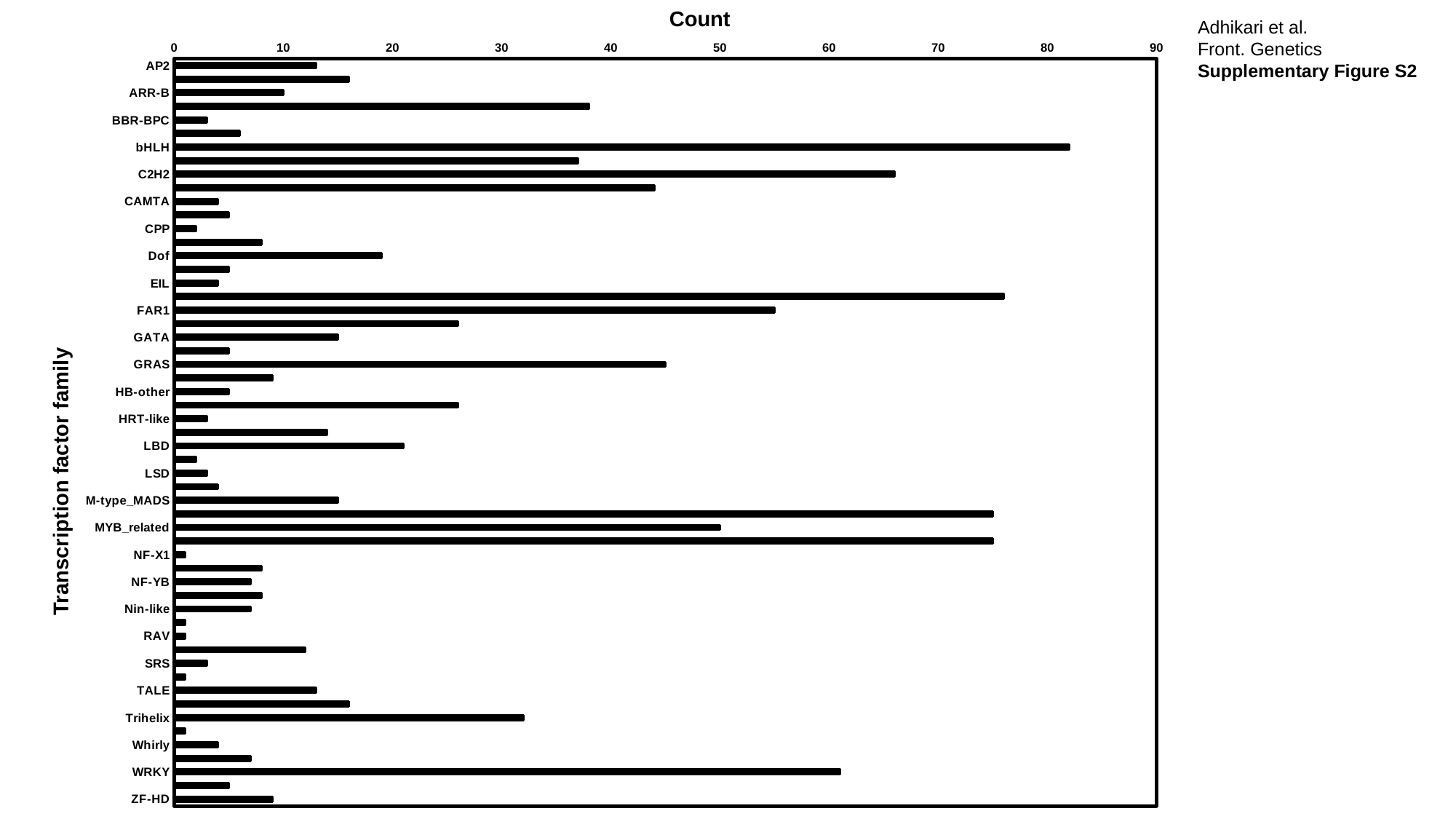

### Chart
| Category | |
|---|---|
| AP2 | 13.0 |
| ARF | 16.0 |
| ARR-B | 10.0 |
| B3 | 38.0 |
| BBR-BPC | 3.0 |
| BES1 | 6.0 |
| bHLH | 82.0 |
| bZIP | 37.0 |
| C2H2 | 66.0 |
| C3H | 44.0 |
| CAMTA | 4.0 |
| CO-like | 5.0 |
| CPP | 2.0 |
| DBB | 8.0 |
| Dof | 19.0 |
| E2F/DP | 5.0 |
| EIL | 4.0 |
| ERF | 76.0 |
| FAR1 | 55.0 |
| G2-like | 26.0 |
| GATA | 15.0 |
| GeBP | 5.0 |
| GRAS | 45.0 |
| GRF | 9.0 |
| HB-other | 5.0 |
| HD-ZIP | 26.0 |
| HRT-like | 3.0 |
| HSF | 14.0 |
| LBD | 21.0 |
| LFY | 2.0 |
| LSD | 3.0 |
| MIKC_MADS | 4.0 |
| M-type_MADS | 15.0 |
| MYB | 75.0 |
| MYB_related | 50.0 |
| NAC | 75.0 |
| NF-X1 | 1.0 |
| NF-YA | 8.0 |
| NF-YB | 7.0 |
| NF-YC | 8.0 |
| Nin-like | 7.0 |
| NZZ/SPL | 1.0 |
| RAV | 1.0 |
| SBP | 12.0 |
| SRS | 3.0 |
| STAT | 1.0 |
| TALE | 13.0 |
| TCP | 16.0 |
| Trihelix | 32.0 |
| VOZ | 1.0 |
| Whirly | 4.0 |
| WOX | 7.0 |
| WRKY | 61.0 |
| YABBY | 5.0 |
| ZF-HD | 9.0 |Count
Transcription factor family
Adhikari et al.
Front. Genetics
Supplementary Figure S2
